# Right fronto-parietal networks mediate the neurocognitive benefits of enriched environments

**DOI:** 10.1093/braincomms/fcac080

**Published:** 2022-03-28

**Authors:** Méadhbh B. Brosnan, Nir Shalev, Jivesh Ramduny, Stamatios N. Sotiropoulos, Magdalena Chechlacz

**Affiliations:** 1 Department of Experimental Psychology, University of Oxford, Oxford, UK; 2 Oxford Centre for Human Brain Activity, University of Oxford, Oxford, UK; 3 Wellcome Centre for Integrative Neuroimaging, University of Oxford, Oxford, UK; 4 Turner Institute for Brain and Mental Health, Monash University, Melbourne, VIC, Australia; 5 School of Psychology, Trinity College Dublin, Dublin, Ireland; 6 Trinity College Institute of Neuroscience, Trinity College Dublin, Dublin, Ireland; 7 Sir Peter Mansfield Imaging Centre, School of Medicine, University of Nottingham, Nottingham, UK; 8 National Institute for Health Research (NIHR), Nottingham Biomedical Research Centre, Queen’s Medical Centre, Nottingham, UK; 9 Centre for Human Brain Health, University of Birmingham, Birmingham, UK; 10 School of Psychology, University of Birmingham, Birmingham, UK

**Keywords:** cognitive ageing, reserve, brain resilience, superior longitudinal fasciculus, diffusion MRI

## Abstract

Exposure to enriched environments throughout a lifetime, providing so-called reserve, protects against cognitive decline in later years. It has been hypothesized that high levels of alertness necessitated by enriched environments might strengthen the right fronto-parietal networks to facilitate this neurocognitive resilience. We have previously shown that enriched environments offset age-related deficits in selective attention by preserving grey matter within right fronto-parietal regions. Here, using neurite orientation dispersion and density imaging, we examined the relationship between enriched environments, microstructural properties of fronto-parietal white matter association pathways (three branches of the superior longitudinal fasciculus), structural brain health (atrophy), and attention (alertness, orienting and executive control) in a group of older adults. We show that exposure to enriched environments is associated with a lower orientation dispersion index within the right superior longitudinal fasciculus 1 which in turn mediates the relationship between enriched environments and alertness, as well as grey and white matter atrophy. This suggests that enriched environments may induce white matter plasticity (and prevent age-related dispersion of axons) within the right fronto-parietal networks to facilitate the preservation of neurocognitive health in later years.

## Teaser

By combining diffusion MRI and cognitive testing, Brosnan *et al*. demonstrate that the microstructure of the right superior longitudinal fasciculus mediates the association between enriched environments and neurocognitive health in older adults. This work suggests that the right fronto-parietal networks may support the phenomenon of cognitive reserve.

## Graphical Abstract

**Graphical Abstract:**
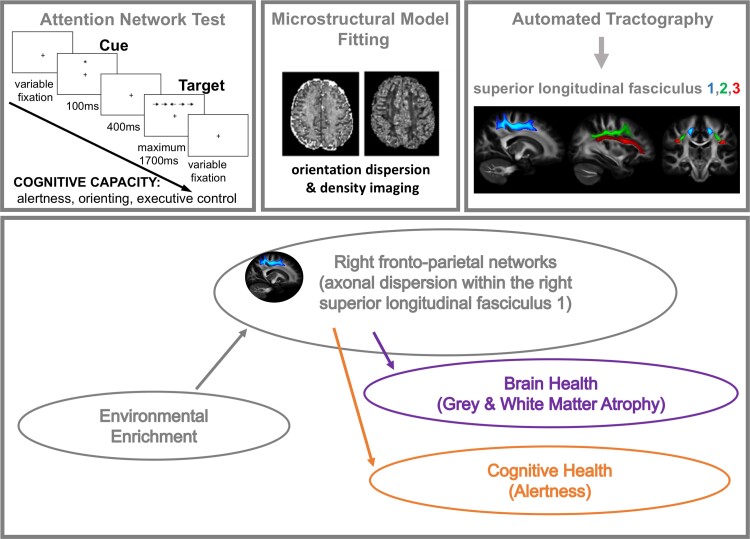

## Introduction

In the foreword to the 2020 World Health Organization (WHO) landmark report on healthy ageing, Dr Tedros Adhanom Ghebreyesus (WHO Director-General) states that ‘Humans now live longer than at any time in history. But adding more years to life can be a mixed blessing if it is not accompanied by adding more life to years’. An integral component of living life to the fullest in our later years is the capacity to maintain adequate cognitive abilities in older age. But human ageing is characterized by a sheer diversity of trajectories, with some older adults maintaining almost youth-like physical and cognitive capacities, and others experiencing frailty, disability and dementia. Multiple modifiable factors across a lifetime have been shown to influence the trajectories of cognitive ageing.^1^ Older adults who have engaged in cognitively and socially enriched environments (EEs) exhibit greater resilience to cognitive decline when faced with neuropathological conditions such as Alzheimer’s disease,^2–5^ a phenomenon referred to as neurocognitive reserve, cognitive reserve or simply reserve.^4,6–8^ The concept of reserve originates from long-standing observations with Alzheimer’s patients whereby higher levels of education attainment delay the onset time of clinical symptoms of the disease and, consequently, cognitive decline.^2,4,9^ However, it is increasingly evident that such benefits to neurocognitive health are not exclusively obtained through education, but are additionally noted for leisure and social activities, and occupational engagements throughout a lifetime.^10,11^ Correspondingly, it has been proposed that a variety of enriched and engaging environments across a lifetime contribute to optimal brain health and to prevent, or at least delay, the onset of dementia later in life.^4,12^ Yet despite compelling, large-scale longitudinal evidence for this phenomenon, we are just beginning to understand the neurobiological basis by which EEs impact the brain to cultivate resilience.

EEs necessitate the continued engagement of several core cognitive processes, including alertness, sustained attention and awareness, all of which rely on the right fronto-parietal networks.^13–20^ Continued activation of these right lateralized networks has been theorized to cultivate and support the neuroprotective phenomenon of cognitive reserve.^20^ The locus coeruleus norepinephrine (LC-NE) alertness system has strong projections to the right fronto-parietal networks,^21–27^ and the strengthening of these networks by EEs over a lifetime is proposed to arise from the continued engagement of the LC-NE system over extended periods.^20^

Numerous studies provide evidence that cognitive experiences (e.g. training or learning new skills) result in experience-dependent brain changes in brain structure in both the grey and white matter (WM) regions (i.e. structural plasticity changes).^28–30^ As such, the beneficial influence of a lifelong exposure to EE on cognitive performance later in life may be understood in terms of structural plasticity processes which may optimize brain functioning and cognitive performance^31,32^ to offset neuroanatomical deficits relating to poor brain health (e.g. structural atrophy).^33^

Our previous work using mathematical models of visual attention,^34^ causal manipulation techniques^34,35^ and voxel-based morphometry^36^ provide increasing support for the proposal that EE may facilitate neuroprotective resilience through specifically impacting the structural [grey matter (GM) volume] and function (lateralized asymmetry of visual processing speed) of right hemisphere fronto-parietal regions (see Van Loenhoud *et al.*^37^ and Robertson *et al.*^19,20^ for a detailed review theorizing right lateralized underpinnings of reserve). Cortical regions within the fronto-parietal networks are connected by WM association pathways comprising three branches of the superior longitudinal fasciculus (SLF) (SLF1, SLF2 and SLF3).^38^ Our recent work suggests that EE offsets age-related deficits in selective attention by preserving GM within right fronto-parietal regions.^36^ Here we test whether the WM microstructure of the right SLF might be cultivated by EE to facilitate better maintenance of attention function in older adults.

WM pathways underpin the efficiency of communication between discrete cortical regions. Physical characteristics, including micro- and macrostructural properties of the WM (including volume, axonal diameter, myelination, fibre coherence and neurite density) influence both the neurophysiological function of the tract and functional connectivity, with subsequent consequences for behaviour.^39–42^ For example, in younger adults, inter-individual differences in the ability to efficiently attend and respond to sensory information vary according to the WM organization of the SLF.^40,42,43^ WM changes associated with the ageing process affect connectivity within neural networks and underlie gradual age-related declines in cognition.^44–47^ In contrast to invasive animal studies and post-mortem anatomical and histological human studies, microstructural properties of WM and age-related WM changes can be non-invasively studied in the living human brain with diffusion MRI (for recent review, see Lerch *et al.*^48^). While diffusion MRI only provides indirect estimates of biological properties of the WM, new developments in acquisition and data modelling increasingly enable more robust and biologically plausible measures of WM microstructural properties.^48–51^ Diffusion tensor imaging (DTI) based on conventional single-shell acquisition protocols and DTI-derived microstructural measures of WM properties, such as fractional anisotropy (FA) and mean diffusivity, have been widely employed to study age-related WM changes.^45–47,52^ However, while DTI-derived measures are indeed sensitive to age-related alterations in WM microstructure, these are non-specific measures, which cannot be attributed to specific changes in tissue microstructure.^53,54^ For example, age-related reduction in FA might be a result of decreased neurite density, changes in fibre orientation dispersion and/or other changes such as in the degree of myelination.^50,54^ In contrast, multi-shell acquisition protocols combined with biophysically plausible models have been shown to provide more specific estimates of the microstructural properties of WM tissue.^48^ One such approach is neurite orientation dispersion and density imaging (NODDI),^50^ which has been previously used to provide detailed accounts of WM changes associated with development, ageing and several neurological disorders.^55–63^ NODDI-derived parameters, intra-cellular volume fraction (ICVF) and the orientation dispersion index (ODI), respectively, measure neurite packing density and dispersion of neurites/axons (an estimate of fibre coherence).^50^ These two parameters are effectively independent features encoded in the FA, both providing a more biologically specific model of observed changes, which could not previously be disentangled from FA measures derived from DTI. For example, previous research suggests that an increase in FA during development is driven by increasing neurite density, while a reduction in FA later in life is driven by the increase in ODI.^58^ Moreover, changes in NODDI-derived parameters have been shown to be more sensitive to ageing and predictive of cognitive performance.^55,64^

In the present study, we use NODDI to examine the relationship between microstructural properties of the SLF, EEs (measured by proxies such as education, professional, leisure and social activities), attentional capacity (representing cognitive processes contributing to reserve) and structural brain health (GM and WM atrophy) in a group of older adults. To assess attention function, we used a well-established task (the Attention Network Test; ANT).^65–67^ The ANT has been developed to measure three partially distinct ‘networks’ supporting attention: *alerting* attention in response to a temporally predictive cue, *orienting* attention in space and exerting *executive control* to resolve conflict and enhance relevant information. To corroborate the proposal that cognitively stimulating environments and experiences across the lifetime strengthen the right lateralized fronto-parietal networks to facilitate neurocognitive health later in life, we hypothesize that EE induces WM plasticity (prevents age-related dispersion of neurites and/or reduction in neurite density) within the right SLF to facilitate better maintenance of attention function (specifically alertness) and superior brain health (less volume atrophy) in older adults.

## Materials and methods

### Participants

A total of 50 older adults (22 males; age range: 65–84; mean ± SD age 73.5 ± 4.7) were recruited for the study, which consisted of behavioural testing [with the Cognitive Reserve Index questionnaire (CRIq) and the ANT task] and an MRI session. All participants were recruited either from the Psychology panel of elderly volunteers, or the Birmingham 1000 Elders group, both established at the University of Birmingham. The two panels of elderly volunteers consisted of adults aged 65 or older who were in good health and had no pre-existing cognitive impairment. All study volunteers had normal or corrected-to-normal vision, had no history of psychiatric or neurological disease and were identified as right-handed. Participants with contraindications to MRI were excluded. The study was approved by the University of Birmingham Ethical Review Committee. All study participants provided written informed consent and received monetary compensation for participation in agreement with the approved ethics protocol.

### Environmental enrichment

To estimate levels of lifelong exposure to EEs, all study participants completed the CRIq.^68^ This is a semi-structured interview, assessing educational attainment, along with the complexity of professional engagements and a wide variety of leisure and social activities. The CRIq measure comprises three subscales (CRI education, CRI working activity and CRI leisure time), and a composite score (overall CRIq).^68^ Each of the three subscales are derived based on both the frequency and duration (in weeks, months or years) of the various activities across lifespan. One of the participants provided incomplete questionnaire data and was removed from the statistical analyses as we were unable to subsequently calculate the CRIq score.

### Attentional capacity

#### The Attention Network Test

**Figure 1:**
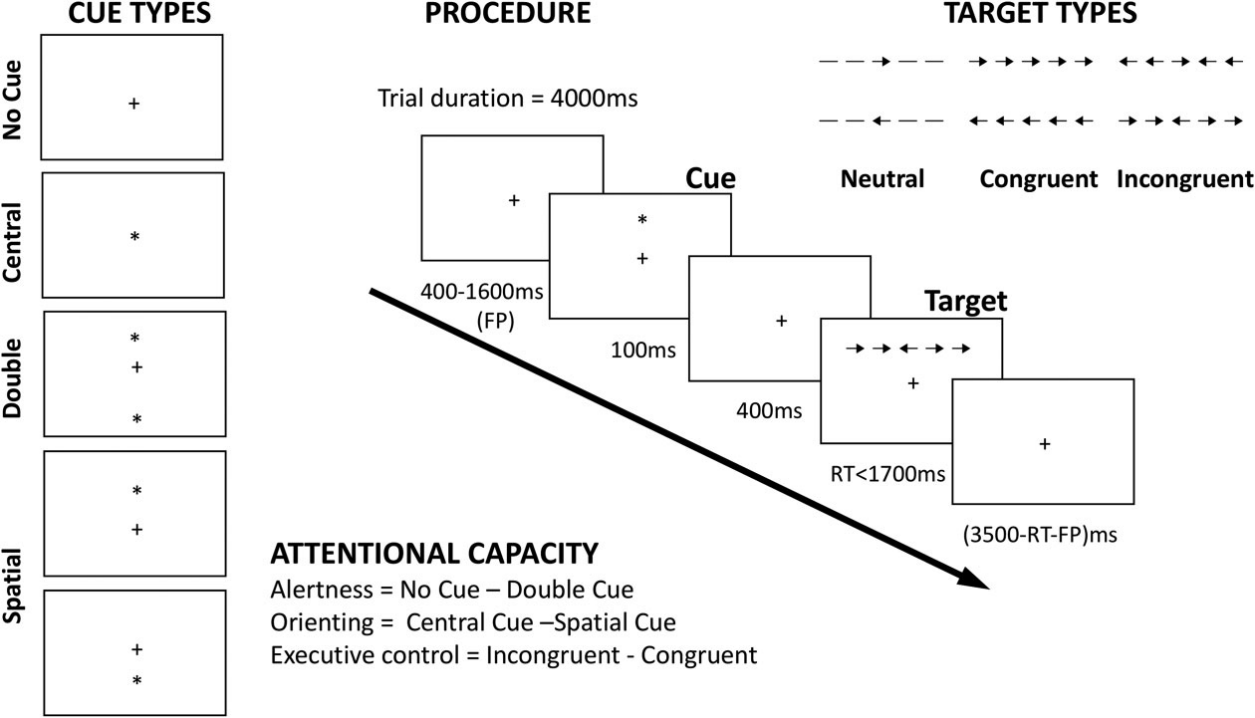
**The ANT.** Illustration of the ANT procedure, including types of target and cue, as well as example of a trial and estimation of attention capacity parameters.

We used the ANT,^65^ which has been employed extensively to study attention among older adults.^69–72^ The task was designed to test the efficiency of the three ‘networks’ supporting attention: *alerting* (achieving and maintaining a state of alertness), *orienting* (the selection of information from sensory input) and *executive control* (resolving conflict among competing response dimensions).^73^ In the task, outlined in Fig. 1, partisans are requested to judge the direction of a central arrow (target) surrounded by four flanking stimuli (two on each side). The target and its surrounding flankers appear in one of two locations, above or below a fixation mark at the centre of the monitor along the central vertical line. The flankers can be neutral flankers (lines with no arrowheads), congruent (arrows pointing in the same direction as the target) or incongruent (arrows pointing in the opposite direction to the central target). Each trial starts with a variable fixation period [400–1600 ms; first fixation period (FP)] and the presentation of the flanked target is preceded by one of four cueing conditions (see Fig. 1). These are as follows: a central cue, where a central warning signal (an asterisk) is presented; a double cue, where two asterisks are presented indicating the two possible locations of the flanked target along the horizontal line; a spatial cue indicating where the target will appear using a single asterisk; and no cue. The cue is always presented for 100 ms and followed by another fixation period of 400 ms. A fixation cross remains at the centre of the screen throughout the trial, with the exception of the central-cue condition where it is replaced for 100 ms by an asterisk. The target surrounded by flankers is always presented either until the participant responds to reaction time (RT) or in the case of no response for a maximum of 1700 ms. In total, each trial lasts for 4000 ms (Fig. 1). Thus, once the target and flankers disappear, the next trial is preceded by a variable fixation interval based on the duration of FP and RT.

#### ANT task procedure

Participants were instructed to attend to the central fixation cross and to use the mouse buttons to indicate the direction of the flanked arrow when presented (i.e. left mouse click for leftward arrowheads and right mouse click for rightward arrowheads). The various conditions (four different cues and three levels of congruency) were randomized within blocks. The task began with 24 practice trials with feedback, followed by three blocks without feedback. Within each block, there were 96 trials, presented in random order. These consisted of cue condition (x4), target locations (x2), target directions (x2), flanker conditions (x3), repetitions (x2). The practise block took ∼2 min, and each subsequent experimental block took ∼5 min to complete.

#### Measuring attentional capacity (ANT scores)

We derived the ANT scores (alertness, orienting and executive control/conflict) as per previously published work^65,74^ by calculating the difference between the mean RT of the different conditions (defined by either cue or target type in the ANT procedure). The alertness score was calculated by subtracting the mean RT in the double-cue condition (i.e. the two warning cues corresponding to the two possible target locations) from the mean RT in the no-cue condition (alerting = RT_no cue_*−*RT_double cue_). As such, the alerting score indicates the degree to which an individual uses the external cues to benefit their reaction times. Larger alerting scores indicate a relative difficulty in maintaining alertness in the absence of an external cue i.e. decreased ability to rely on internal (or intrinsic) alertness.^74^ However, it is possible that larger alertness scores might instead indicate a more efficient use of the cue. One way to dissociate the two is by testing for a correlation between the *alerting* score and the mean reaction time from the no-cue condition. A positive association would imply that participants who relied more on the alerting cue to facilitate performance were slower, thereby indicative of decreased intrinsic alertness capacities. The orienting is calculated by subtracting the mean RT in the spatial-cue condition from the mean RT in the central-cue condition (orienting = RT_central cue_−RT_spatial cue_). The spatial cue, which is always valid, provides information about subsequent target location and thus facilitates orienting attention before target arrival. As such, the orienting score reflects the difference between responses to targets that follow spatially predictive and non-predictive cues. Larger orienting scores indicate a better capacity to orient attention and select sensory input.^65^ Finally, the executive control score is calculated by subtracting the mean RT in the congruent condition from the mean RT in the incongruent condition across all cue types (executive control/conflict = RT_incongruent_ −RT_congruent_). The conflict score represents the capacity to resolve response conflict to targets that appear among distractors. The larger conflict score indicates less ability (difficulty) in resolving conflict.^74^ We present descriptive statistics and estimate the Pearson correlations among the four task indices: alerting, orienting, conflict and mean reaction time (RT).

### Fronto-parietal microstructure

#### MRI data acquisition

T_1_-weighted scans (MPRAGE with spatial resolution 1 × 1 × 1 mm^3^) and multi-shell diffusion-weighted images were acquired at the Birmingham University Imaging Centre using a Philips 3T Achieva MRI system with a 32-channel head coil. The multi-shell diffusion acquisition comprised a single-shot EPI, 2 × 2 × 2 mm^3^, 5 × *b* = 0 s/mm^2^, 50 × *b* = 1000 s/mm^2^, 50 × *b* = 2000 s/mm^2^ plus 5 × b = 0 s/mm^2^ phase encoding-reversed to correct for susceptibility-induced artefacts.^75^ T_1_-weighted scans were acquired with the following parameters: 176 slices, TR = 7.5 ms, TE = 3.5 ms and flip angle = 8°. Diffusion-weighted scans were acquired with the following parameters: 56 slices, TR = 9000 ms, TE = 81.5 ms and flip angle = 90°. All scans were visually inspected during and after the acquisition to ensure that there have not been any artefacts associated with excessive head movement and signal dropouts. All data passed this quality assessment.

#### T_1_-weighted data pre-processing and total grey matter volume estimation

T_1_-weighted scans were pre-processed using the UK Biobank T_1_-weighted pipeline,^76^ which was used to correct for bias fields, apply skull-stripping and align data to the MNI152 standard space [a non-linear registration to MNI152 using FMRIB's non-linear image Registration Tool (FNIRT), was applied^77,78^], before segmenting the T_1_ images into different tissue classes, i.e. GM, WM and CSF using FMRIB's Automated Segmentation Tool^79^. These data were subsequently used to calculate total GM and total WM volume in mm^3^, normalized for head size using SIENAX package.^80^

#### Diffusion data pre-processing, microstructural model fitting and SLF tractography

**Figure 2:**
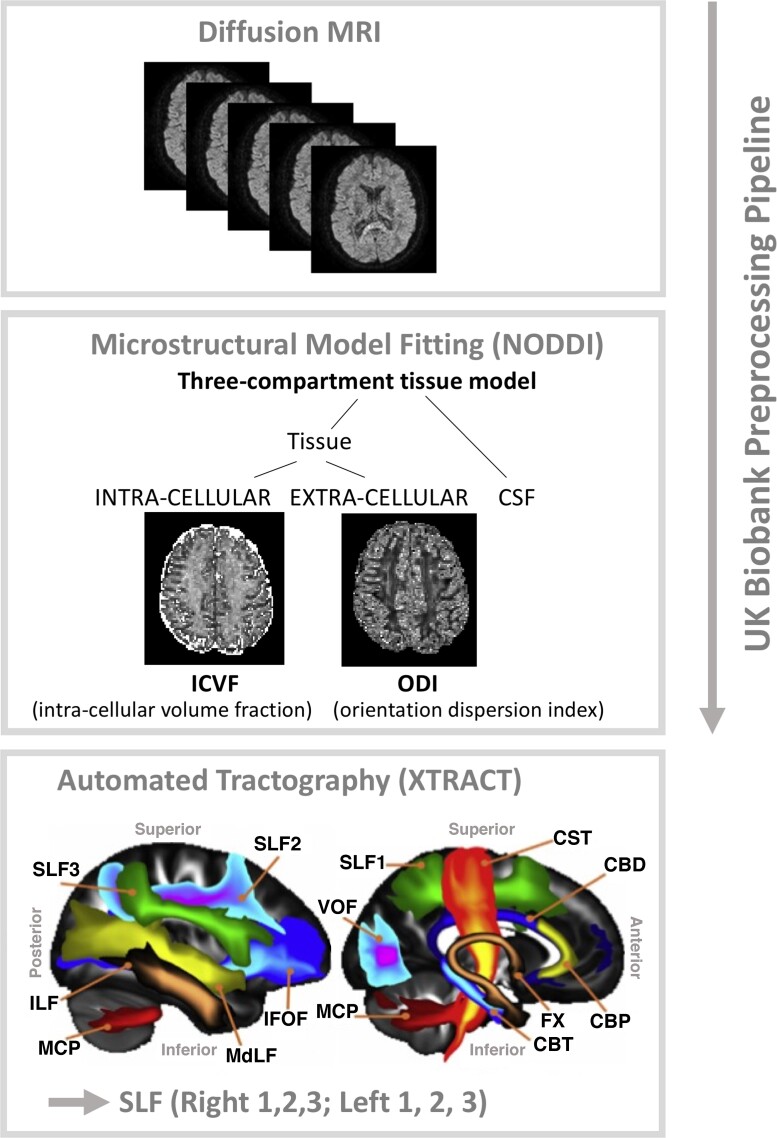
Overview of the diffusion MRI data analysis pipeline.

Diffusion-weighted scans were pre-processed using the UK biobank pipeline^76^ (Fig. 2). The diffusion-weighted pre-processing was applied to correct for susceptibility-induced distortion, eddy-current distortion and participant movement induced distortions using the EDDY toolbox^81^ and to obtain transformations of the diffusion to structural and standard space. Specifically, the corrected dMRI data were linearly registered to structural space, using a rigid body transformation (FMRIB's Linear Image Registration Tool,^82,83^). Structural data were non-linearly registered to standard space using FNIRT. Concatenating the two provided a non-linear transformation from diffusion to standard space. Subsequently, we applied the NODDI model to the multi-shell EDDY-corrected diffusion data^50,84^ using the CUDA Diffusion Modelling Toolbox (https://users.fmrib.ox.ac.uk/∼moisesf/cudimot/)^85^ to estimate voxel-wise microstructural parameters, including ICVF, and ODI. In addition, a diffusion tensor model^86^ was fitted to low *b*-value (*b* = 1000 s/mm^2^) shells of the EDDY-corrected diffusion data to obtain FA maps for each participant. Next, we performed automated probabilistic tractography using predefined protocols for identifying major WM tracts in the left and right hemispheres, as described in FSL’s XTRACT tool (https://fsl.fmrib.ox.ac.uk/fsl/fslwiki/XTRACT^87,88^). Prior to running XTRACT, we fitted the crossing fibre model (FSL’s BEDPOSTX^89^) to each subject’s data to estimate up to three fibre orientations per voxel and ran non-linear transformations to the MNI152 standard space. XTRACT automatically reconstructs a set of predefined white matter pathways, including the three branches of the SLF (SLF1, 2, 3). These three tracts were reconstructed and used for the purpose of data analysis. The SLFs’ tract probability density maps, normalized by the total number of valid streamlines, were thresholded at 0.1% and binarized to produce a tract mask for each tract in standard space. Finally, for each participant, we calculated a set of image-derived phenotypes, characterizing different microstructural properties of the three branches of SLF. Specifically, for each tract (left and right SLF1, 2, 3), and for each DTI (FA) and NODDI (ODI, ICVF) parameter, the weighted mean value (i.e. the mean weighted by the tract probability in each voxel) of the parameters within given tract was calculated. We checked all neuroimaging measures for outliers, which were defined as any scores greater or less than three times the inter-quartile range. Based on these criteria, two participants were excluded. Subsequently, all statistical analyses as described below were conducted with a sample of *N* = 47 after the removal of these two outliers and the participant with missing CRIq data.

### Statistical analyses

To assess whether environmental enrichment was differentially associated with the hemisphere-specific microstructural organization of the SLF, we modelled EE (the composite CRIq score) as a function of three separate microstructural estimates: ODI, ICVF and FA within the three SLF branches in each hemisphere using the following linear regression approach. For each model, age was entered as a nuisance covariate in the first step, and then the ODI estimates of the six SLF branches (i.e. branches 1–3 for both hemispheres) were entered in the model using a stepwise approach. These analyses revealed that ODI (within the right SLF1) was particularly sensitive to the impact of EE. In the analyses above, EE was estimated using the composite CRIq score. To identify whether specific types of enrichment were driving the observed effect of EE on the right SLF1, a follow-up analysis of the three subscales (education, occupational and leisure engagements) was performed.

To investigate whether ODI within the SLF was associated with the degree of GM and WM volume atrophy (GMVa and WMVa, respectively) in the older individuals’ brains, we modelled total GM and total WM volume (normalized for head size), as a function of ODI within the three SLF branches in each hemisphere. Again, age was entered as a nuisance regressor in step 1 for both models, and the ODI estimates for all six SLF branches were entered into each model with a stepwise approach. These analyses identified a specific association between both GMVa and WMVa with ODI within the right SLF1. We subsequently tested whether a causal association existed between EE, ODI within the right SLF1 and brain atrophy (separately GMVa and WMVa) using bootstrapped mediation analyses. For this, bootstrapped mediation analyses (5000 samples) were performed using the PROCESS computational toolbox.^90,91^ More specifically, the plausibility of a causal model was investigated, whereby EE (predictor variable *X*) causally influenced white matter microstructure (ODI) of the right SLF1 (mediator variable *M*), to in turn exert a causal influence over GMVa and WMVa in the older adults (outcome variables *Y*). The confidence intervals (CIs) reported for the indirect effect are bootstrapped CIs, based on 5000 samples, and are considered significant when they do not contain zero.

Finally, to determine whether the right SLF1 showed a meaningful relationship to attentional capacity, we next modelled ODI within this tract as a function of the three ANT scores (alertness, orienting and executive control) using a stepwise regression model, again with age as a nuisance covariate in the first step of the model. As we observed an association between ODI within the right SLF1 and alertness, we subsequently tested whether a causal association existed between EE, ODI within the right SLF1 and alertness using bootstrapped mediation analysis as described above (causal mediation model EE-> rSLF1 -> alertness). To explore which aspect of EE was driving our effects, a follow-up mediation analysis with the three subscales (education, occupational and leisure engagements) of CRIq was performed.

Before the analyses as described previously, all data sets were checked to ensure no statistical assumptions were violated. The normality of residuals and heteroskedascticity were tested for all analyses by visual inspection of the residuals, and formal analysis using the Shapiro–Wilks test of normality. Statistical analyses were conducted using a combination of SPSS software version 27 (IBM SPSS Statistics, NY, USA) and MATLAB r2021a (The MathWorks, Natick, MA, USA).

With null hypothesis significance testing, non-significant effects should not be interpreted as support for the null hypothesis.^92^ To address whether non-significant findings represented support for the null hypotheses and to verify the results from our frequentist statistics, we supplemented the above analyses using the Bayesian inference approach. For these analyses, we used JASP software (version 0.11.1, JASP Team 2019)^93^ with the Bayesian information criterion (BIC)^94^ to compare model fits. Model priors were set using the default settings in JASP (beta-binomial *a* = 1, *b* = 1). We report the *P*(M|data) values indicating the posterior probability of each model (i.e. the probability of each model after observing the data) and consider all values >0.05. We additionally report BF_M_ values which compare a given model against all other models, i.e. the factor by which the odds in favour of a given model have increased having observed the data. BF_M_ values above 1 indicate the strength of evidence in favour of the alternative and values below 1 indicate strength of evidence in favour of the null model.^95,96^

### Data availability statement

Participant data will be made available to researchers upon request.

## Results

### Attentional capacity: performance on the ANT

The overall mean accuracy was high (mean accuracy 98%; SD = 0.02%; range: 9–100%) indicating that participants did not have any difficulty completing the task. Our analysis focused on RT-based indices measuring individual differences in attentional capacity, i.e. three network scores used to represent the efficiency of the alerting, orienting and executive control.^65,74^Table 1 summarizes RT data averaged for each target and cueing condition.

**Table 1:** The mean RT (standard error) for each target and cueing condition

	Cue type			
Target type	No cue (ms)	Double (ms)	Central (ms)	Spatial (ms)
Neutral	717 (15.33)	665 (13.46)	693 (13.85)	637 (12.76)
Congruent	735 (16.21)	680 (12.54)	707 (15.10)	643 (13.44)
Incongruent	857 (19.02)	829 (19.48)	838 (18.05)	762 (16.90)

**Figure 3:**
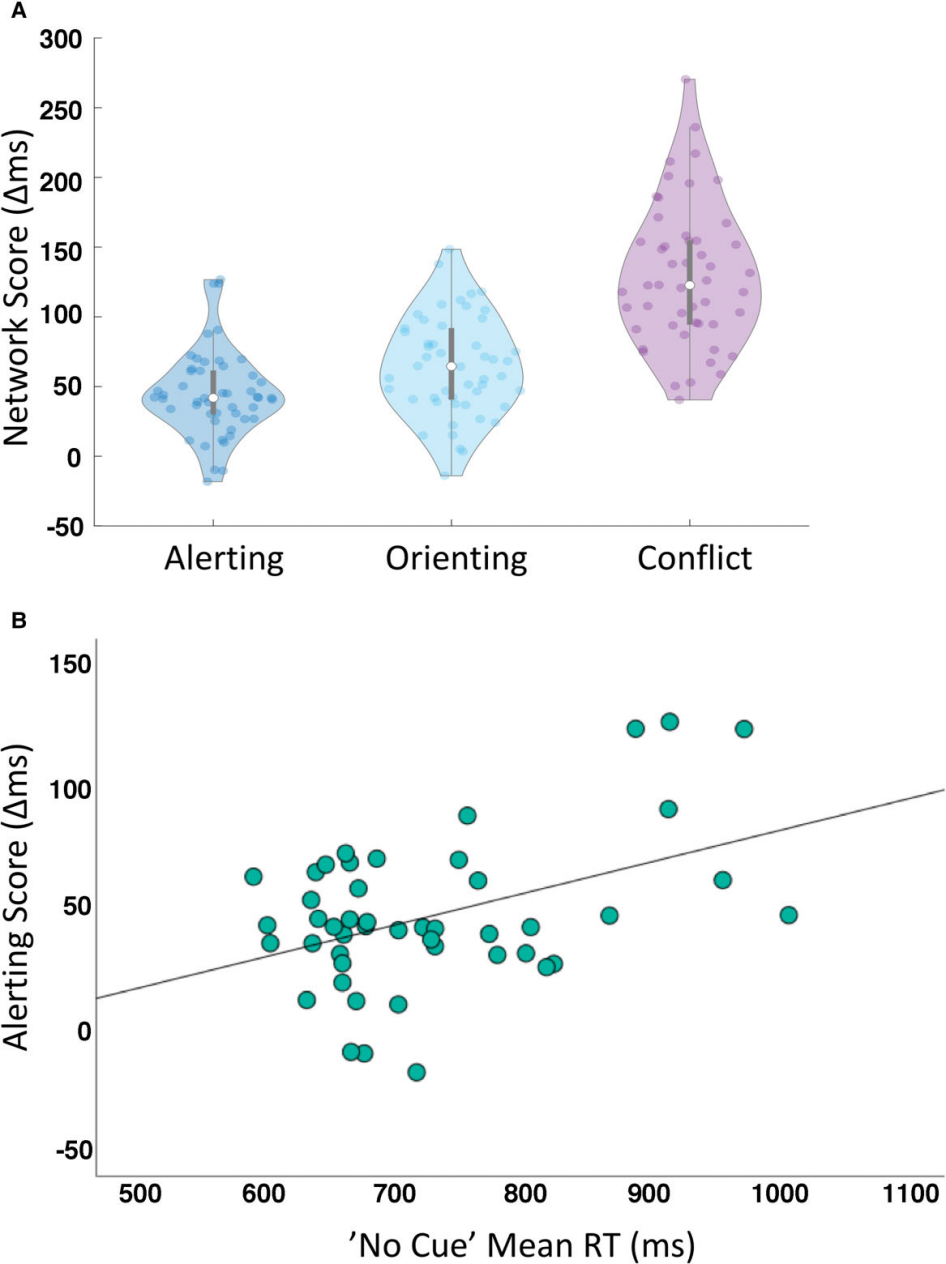
**Performance on the ANT (derived *network* scores; alerting, orienting and conflict).** (**A**) The alerting, orienting and conflict network scores based on the ANT performance are plotted to illustrate attentional capacity distribution in the studied group of (*N* = 47) older adults. (**B**) The scatter plot for the correlation between the alertness network score and ‘no cue’ mean RT (*r* = 0.56, *P* < 0.0005).

The distribution of the network scores is depicted in Fig. 3A. The scattered data points illustrate that the majority of the observations were positive, thus indicating that in this cohort of older adults, most participants (i) benefitted from the alerting cue (i.e. were faster when presented with a cue); (ii) showed a good capacity to orient attention, i.e. benefitted from a spatial cue (orienting attention)s before the arrival of target; and (iii) were better at judging congruent targets, compared with incongruent i.e. had decreased ability (difficulty) in resolving conflict.

As suggested in the ‘Materials and methods’ section, the interpretation of the ANT alertness measure is not straightforward as a larger alerting score could be indicative of either decreased ability to rely on internal alertness or more efficient use of a cue. We tested the correlation between the alertness score and the mean RT in the ‘no cue’ condition and found a significantly high correlation (*r* = 0.56, *P* < 0.0005; Fig. 3B). Accordingly, people who benefitted more from the alerting cue were also relatively slow when there was no cue. This is in line with the former interpretation of the alertness index such that in our sample, higher scores were associated with a lower level of internal alertness.

The mean network scores (effect), mean RT and correlations between these indices are shown in Table 2. The correlation analyses were conducted to examine independence of the attentional capacity measures. As in originally published ANT paper^65^ reporting no significant correlations between network scores, we found no correlations between alerting, orienting and executive control scores indicating the independence of estimated measures of alertness, orienting and executive control. The mean RT correlated with the alerting and conflict scores.

**Table 2:** The mean network scores (effect), mean RT (standard error) and correlations between attention networks

	ANT score	Alerting	Orienting	Conflict
Alerting (Δms)	45.1 (4.34)			
Orienting (Δms)	64.7 (5.04)	*r* = 0.08		
Conflict (Δms)	129.8 (7.18)	*r* = 0.24	*r* = −0.12	
Mean RT (ms)	730 (14.6)	*r* = 0.39**	*r* = 0.32	*r* = 0.51***

*** *P* < 0.0005.

** *P* < 0.005.

### EE mitigates neurite (axonal) dispersion in the right SLF1

**Figure 4:**
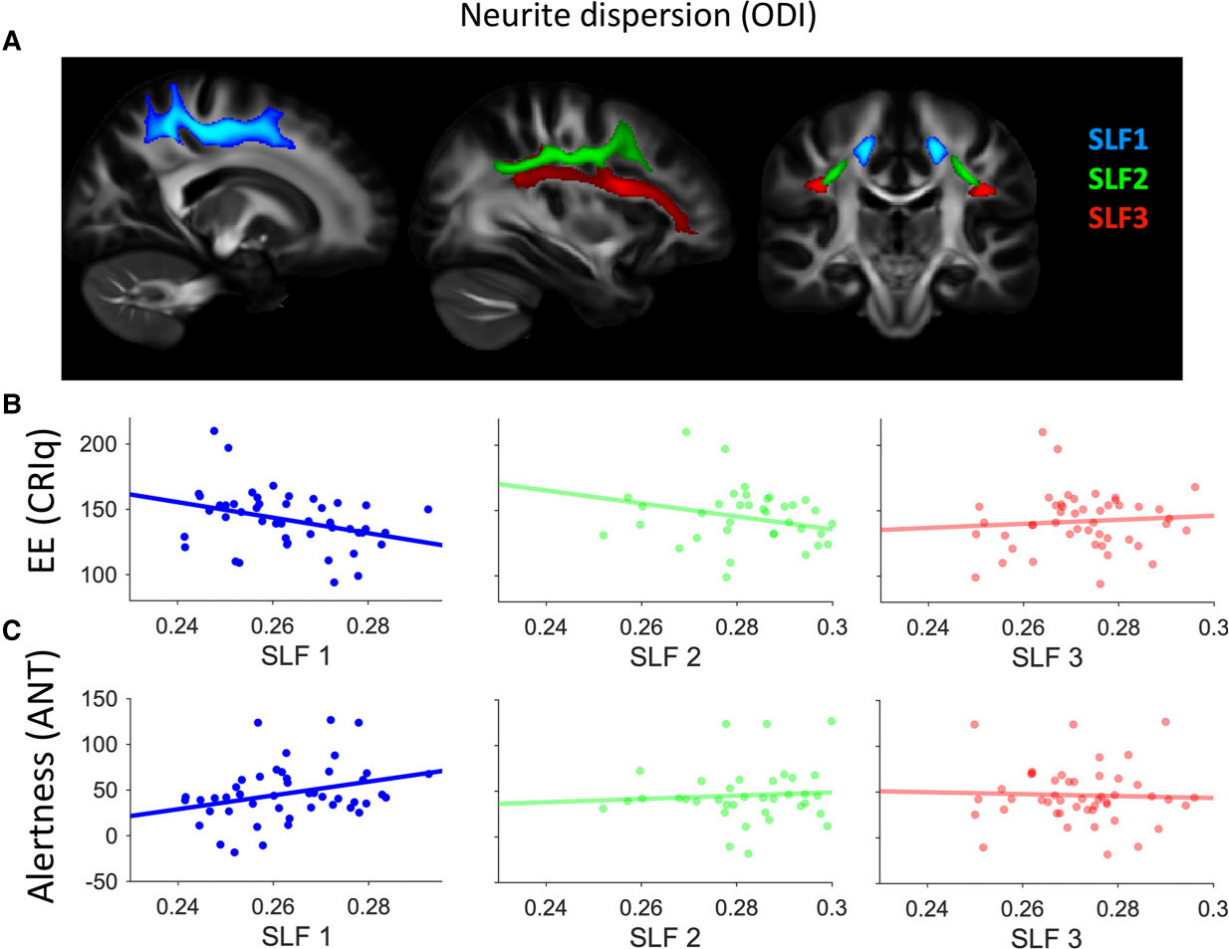
**Neurite dispersion (ODI) in the right SLF1 is associated with EE and with alertness score.** (**A**) XTRACT reconstructed SLF branches. The relationship between ODI within the right SLF branches (SLF1, 2 and 3) and EE (**B**) and alertness (**C**) in the studied group of (*N* = 47) older adults. Only right SLF1 showed a statistically significant relationship to EE (*t* = 2.45, *P* = 0.02) and alertness (*t* = 2.17, *P* = 0.04).

We modelled EE (the composite CRIq score) as a function of ODI within the SLF branches (SLF1, 2 and 3) in each hemisphere (Fig. 4). Age was entered as the first step in the model and offered no significant improvement in model fit over the intercept only model [*R^2^*_change_ = 0.02, *F*_change_ = 0.94, *P F*_change_ = 0.34, unstandardized beta = 0.67, 95% CI (−0.71 2.04), SE = 0.68, standardized beta = 0.14, *t* = 0.97, *P* = 0.34]. Next, ODI estimates for each of the six SLF branches were entered into the model using a stepwise approach. This model offered a significant improvement in fit [*R*^2^_change_ = 0.12, *F*_change_ = 5.86, *P F*_change_ = 0.02, unstandardized beta = −588.41, 95% CI (−1078.12 to −98.69), SE = 252.99, standardized beta = −0.339, Cohen's *F^2^* = 0.16) and led to the inclusion of the right SLF1 (*t*=−2.42, *P* = 0.02, Fig. 4), and exclusion of all other SLF branches (all *t* > −1.50, *P* > 0.14], indicating that greater EE was associated with less dispersion of neurites (lower ODI) within this tract. To obtain accurate parameter estimates of this model, not influenced by other uninformative variables, we modelled EE directly as a function of the right SLF1 and report the results in Table 3. We next assessed whether EE affects neurite density (as measured by ICVF) of the SLF using a similar modelling approach, this time with ICVF within the SLF branches as predictor variables. We found no evidence that EE was associated with altered neurite density (ICVF) in any of the SLF branches (all *t* < 1.10, *P* > 0.28), suggesting that EE specifically altered neurite dispersion and not neurite density within the right SLF1. Finally, using the same modelling approach, we assessed whether EE varied as a function of FA. We found no evidence that EE was associated with altered FA within any of the SLF branches (*all t* < 0.33, *P* > 0.13).

**Table 3:** Neurite dispersion of the right as a function of EE, GMVa, WMVa and alertness

	Unstand. *β*	Std error	Stand. *β*	*T*	*P*-value	95 % CI	Cohen’s *F*^2^
EE	−0.000198	.000081	−0.34	−2.45	0.02	(0.00036 to −0.000035)	0.13
GMVa	−1359.71	457.98	−0.41	−2.97	0.005	(−2282.13 to −437.30)	0.20
WMVa	−1416.16	578.64	−0.34	−2.45	0.02	(−2581.61 to −250.72)	0.13
ANT alerting	0.00013	0.00006	0.31	2.17	0.04	(0.000009−0.000242)	0.10

Cohen’s *F*^2^ calculated as [*R*^2^/(1−*R*^2^)]. The reported parameter estimates here are from separate models as described in the main text.

To address whether the non-significant findings reported above represented support for the null hypotheses and to verify the results from our frequentist statistics, we supplemented the above analyses using the Bayesian inference approach, testing the associations between EE and white matter microstructure (ODI, ICVF and FA) of the three SLF branches in each hemisphere and age. Consistent with the frequentist statistics, the best-fitting model of EE from the BIC analyses with ODI for all six SLF branches included ODI within the right SLF1 [*P(M|*data) = 0.15]. After observing the data, the odds of including ODI of the right SLF1 in a model of EE increased by a factor of 9.34 (BF*_M_* = 9.34). The next best-fitting model contained ODI of the right SLF2 [*P(M|*data) = .12; BF*_m_* = 7.5], suggesting that this right hemisphere pathway additionally varies according to EE. The *P*(*M*|data) of all other models was *≤*0.05.

Repeating the same Bayesian model with FA of the six SLF branches indicated only one model which slightly improves over and above the null model; FA of the right SLF2 [*P(M|*data) = 0.054]. More specifically, the odds of including FA of the right SLF2 in a model of EE increased by a factor of 3.15 after observing the data (BF_M_ = 3.15). The *P*(*M*|data) of all other models were ≤0.03. Finally, when modelling EE as a function of the ICVF metrics of the SLF, the *P*(*M*|data) of all models was ≤0.025.

### The effect of EE on the right SLF1 is driven by professional engagement

Having observed that EE was associated with altered neurite/axonal dispersion within the right SLF1, we next asked the question of whether this effect was driven by any specific facet of enriching activities. Our assessment of EE (the CRIq) comprised three subscales; leisure, work and education. As such, we explored the direct association between the right SLF1 and all three subscales using a model with the right SLF1 as the dependent variable, age as a nuisance predictor in the first stage of the model and the three CRIq subscales as predictors in the second stepwise stage of the model. Age offered no significant improvement in fit [*R^2^*_change_ = 0.001, *F*_change_ = 0.02, *P F*_change_ = 0.88, unstandardized beta = −6.22e^5^, 95% CI (0–0), SE = 0, standardized beta = −0.02, *t* = −0.16, *P* = 88]. However, there was a specific association between the work subscale of the CRI and the right SLF1 such that greater occupational complexity and professional engagements were associated with decreased dispersion of neurites (ODI) in the older adults [*R^2^*_change_ = 0.22, *F*_change_ = 12.26, *P F*_change_ = 0.001, unstandardized beta = 0.000, 95% CI (0.000–0.000), SE = 0.000, standardized beta = −0.47, *t* = −3.50, *P* = 0.001, Cohen’s *F^2^* = .28]. No such associations were observed for either leisure activities or education (both *t* < 0.05, *P* > 0.95).

Follow-up analyses comparing model fits using the Bayesian inference criterion indicated that the best-fitting model for ODI of the right SLF1 contained the work subscale of the CRIq (*P(M*|data) = 0.43). More specifically, after observing the data, the odds of including CRIq work in a model of ODI of the right SLF1 increased by a factor of 14.44 (BF*_M_* = 14.44). These odds were over five times the two next best-fitting models, which included a combination of work with education [Model 2: *P*(*M|*data = 0.09; BF*_M_* = 2.7] and work with leisure engagements [Model 3: *P*(*M|*data = 0.09; BF*_M_* = 2.7]. Model 4 contained a combination of all variables [i.e. work, education, leisure and age; *P*(*M|*data = 0.08; BF*_M_* = 0.35]. All other models had a posterior probability *≤*0.05. This pattern of results suggests that in this cohort work is, by far, the strongest predictor of ODI in the right SLF1.

### The right SLF1 mediates the association between EE and Structural Brain Health (grey and white matter atrophy)

To test whether ODI of the SLF relates to the structural health of the ageing brain, both grey matter and white volume (both normalized by head size) were modelled separately as a function of age and the six SLF tracts (SLF 1–3, left and right hemisphere). For grey matter volume atrophy (GMVa) when age was entered in the first step of the model, it offered no significant improvement in model fit over the intercept only model [*R*^2^_change_ = 0.01, *F*_change_ = 0.47, *P F*_change_ = 0.5, unstandardized beta = −0.91, 95% CI (−3.60 to 1.78), standardized beta = −0.10; SE = 1.33, *t* = −0.68, *P* = 0.50]. Next, ODI estimates for each of the six SLF branches were entered into the model of GM volume using a stepwise approach. This model offered a significant improvement in fit (*R^2^*_change_ = 0.17, *F*_change_ = 8.85, *P F*_change_ = 0.005) and led to the inclusion of the right SLF1 [unstandardized beta = −1368.67, 95% CI (−2296.17 to −441.57), standardized beta = −0.41 ; SE = 459.87, *t* = −2.98, *P* = 0.005, Cohen’s *F^2^* = 0.22], and exclusion of all other SLF branches (all *t* < 0.55, *P* > 0.58), indicating that less dispersion of neurites (i.e. lower ODI) within the right SLF1 was associated with less GMVa (i.e. greater total GM volume). To obtain accurate parameter estimates of this model, not influenced by other uninformative variables, we modelled GMVa directly as a function of the right SLF1 and report the results in Table 3.

For WMVa, again age was entered as the first step in the model and offered no significant improvement in model fit [*R^2^*_change_ = 0.01, *F*_change_ = 0.34, *P F*_change_ = 0.56, unstandardized beta = −0.95, 95% CI (−4.26 to 2.35), standardized beta = −0.09, SE = 1.64, *t* = −0.58, *P* = 0.56]. Adding the ODI estimates for the six SLF branches using a stepwise approach offered a significant improvement in fit (*R^2^*_change_ = 0.12, *F*_change_ = 5.99, *P F*_change_ = 0.02) and again led to the inclusion of the right SLF1 [unstandardized beta = −1425.22, 95% CI (−2598.94 to −251.5), standardized beta = −0.35; SE = 582.39, *t*=−2.45, *P* = 0.02, Cohen’s *F^2^* = 0.15] and exclusion of all other SLF branches (all *t* < 0.45, *P* > 0.41), indicating that less dispersion of neurites (lower ODI) of the right SLF1 was associated with less WMVa (i.e. larger total WM volume). To obtain accurate parameter estimates of this model, not influenced by other uninformative variables, we modelled WMVa directly as a function of the right SLF1 and report the results in Table 3.

To bolster these results, we employed Bayes Factor analyses. When modelling GMVa as a function of ODI for all six SLF branches and age, the best-fitting model was ODI of the right SLF1 [*P(M|*data = 0.43; BF*_m_* = *41.26*], indicating that after observing the data, the odds of including neurite dispersion of the right SLF1 in a model for GMVa increased by a factor of 41.26. All other models had a posterior probability of *<*0.05. When the same analysis was repeated with WMVa, the best-fitting model was again ODI of the right SLF1 *P*(*M*|data = 0.21, BF*_m_* = 7.75), indicating the odds of including right SLF1 ODI in a model for WMVa increased by a factor of 7.75. All other models had a posterior probability of <0.05.

**Figure 5:**
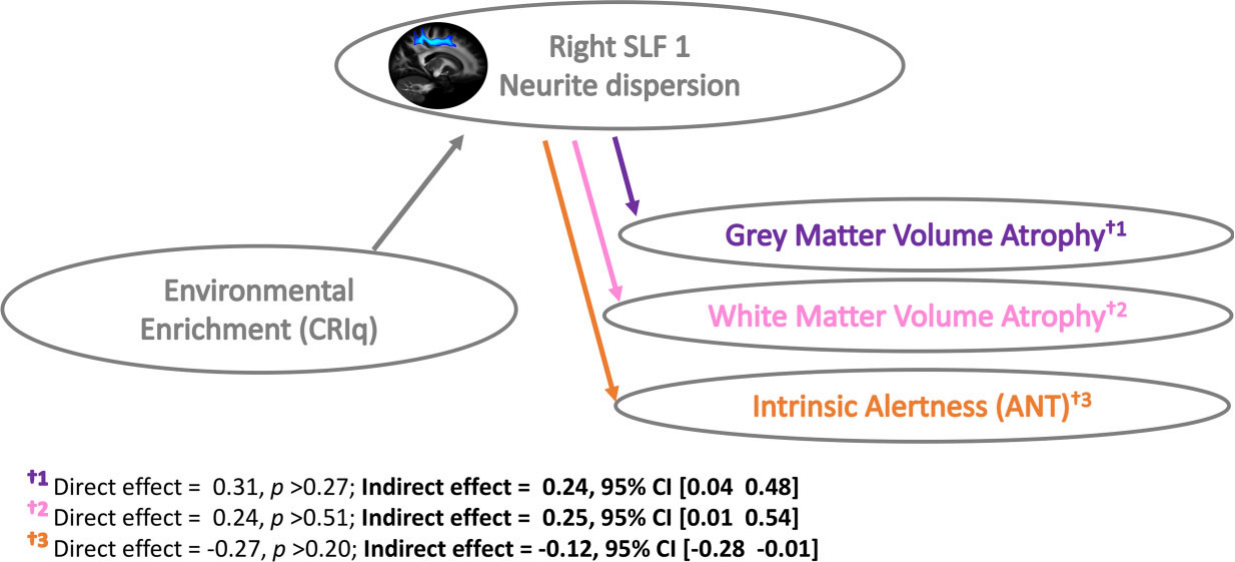
**The right SLF1 mediates the association between EE and neurocognitive health.** Causal mediation models showing an indirect relationship between EE and Structural Brain Health (grey and white matter atrophy), as well as EE and alertness in older adults (*N* = 47), which is mediated by neurite dispersion within the right SLF1.

Our results so far indicate that EE is associated with less neurite dispersion (ODI) of the right SLF1 and that less ODI within this specific tract is associated with less atrophy in both GM and WM. This raises the possibility that a causal relationship may exist such that the extent to which EE positively impacts structural brain health (i.e. offsets white and GMVa) is dependent, at least partly, on the degree to which EE has altered WM properties of the right SLF1. To formally test this hypothesis, we ran two causal mediation models (EE → ODI rSLF1 → GMVa; EE → ODI rSLF1 → WMVa). Bootstrapped mediation analyses showed an indirect effect for both GMVa [direct effect = 0.31, *P* = 0.28; indirect effect = 0.23, 95% CI (0.04–0.48)] and WMVa [direct effect = 0.24, *P* = 0.51; indirect effect = 0.25, 95% CI (0.01–0.54)]. This indicates that the degree to which EE positively mitigates GM and WM atrophy of the ageing brain is dependent in part, on the degree to which EE has altered neurite dispersion properties of the right SLF1 (Fig. 5).

### Neurite dispersion in the right SLF1 is associated with an alertness score

The results thus far demonstrate that EE facilitates superior maintenance of neurite (axonal) dispersion in older adults within the right SLF1. This suggests that the plasticity of the right SLF1 might be specifically sensitive to the beneficial effects of environmental enrichment on ageing brain health. To assess whether the right SLF1 showed a meaningful relationship to alertness, here we modelled this tract as a function of the three subscales of the ANT; alertness, orienting, conflict, see Fig. 4C). Age was entered as the first step in the model and offered no improvement on the intercept only model [*R*^2^_change_ = 0, *F*_change_ = 0.02, *P F*_change_ = 0.88, unstandardized beta = 0, 95% CI (−0.001 to 0.001), standardized beta = −0.02, SE = 0, *t*=−0.16, *P* = 0.88]. Next, we modelled the right SLF1 as a function of the three ANT subscales using a stepwise approach. This led to a significant improvement in model fit (*R*^2^_change_ = 0.09, *F*_change_ = 4.59, *P F*_change_ = 0.04) with neurite dispersion of the right SLF1 uniquely associated with the alertness [unstandardized beta = 0.000, 95% CI (0.000–0.000), SE = 0, standardized beta = 0.31, *t* = 2.14, *P* = 0.04, Cohen’s *F^2^* = 0.11] but not orienting (*t* = −1.00, *P* = 0.32) or conflict (*t* = −1.63, *P* = 0.11) components of the task. To obtain accurate parameter estimates for the relationship between neurite dispersion of the right SLF1 and ANT alertness, we modelled the direct association without other uninformative signals and report the results in Table 3.

Follow-up Bayesian factor analyses revealed two models with a posterior probability of >0.05 after having observed the data. Of these, the best-fitting model included only the alertness score of the ANT [*P(M|*data) = 0.19], such that the odds of including ANT alertness in the model of the SLF1 were more than 4.5 times as high after observing the data (BF*_m_* = 4.51). In contrast, both the posterior probability and the odds of the next best model were lower (model including the alertness and executive control subscales; *P*(*M|*data) = 0.08, BF*_m_* = 2.41).

### The right SLF1 mediates the association between EE and alertness

The findings thus far suggest that a lifetime of EE is associated with reduced neurite (axonal) dispersion within the right SLF1 in older adults. In addition, we observed that reduced right SLF1 neurite dispersion is consequential to cognition, such that it facilitates better maintenance of internal alertness in advanced age (Fig. 4). This raises the possibility of a causal association between these factors such that EE facilitates alertness by impacting the plasticity of the right SLF1. To test this hypothesis, we ran a causal mediation model; EE → ODI rSLF1 → Alertness (Fig. 5). Bootstrapped mediation analyses (5000 samples) revealed a significant mediation such that EE was associated with superior WM microstructure (reduced neurite dispersion) within the right SLF1 which, in turn, facilitated better internal alertness. We note there was no direct relationship between EE and alertness, suggesting that the association between these two variables is dependent on WM properties of the right SLF1 [direct effect = −0.27, *P* = 0.21; indirect effect = −0.1172, 95% CI (−0.2837 to −0.0098)]. In accordance with the findings above which highlighted a privileged association between occupational engagements and ODI of the right SLF1, repeating the mediation model with the work subscale (as opposed to the composite CRIq score) additionally resulted in a significant mediation effect (direct effect = −0.1924, *P* = 0.44; indirect effect = −0.1777, 95% CI (−0.4018 to −0.0185)].

## Discussion

In the present study, we examined how microstructural properties of the SLF varied according to exposure to EEs to subsequently influence markers of neurocognitive health in older adults. Our findings indicate that greater exposure to EEs across a lifetime is associated with reduced axonal dispersion within the right SLF1 in older adults. This in turn mitigates declines in structural brain health (assessed via GMVa and WMVa) and intrinsic alertness (captured with the well-validated ANT test). To the best of our knowledge, this is the first study providing direct evidence linking microstructural properties of a long-range association pathway within the right fronto-parietal networks (the right SLF1) to behavioural markers of neurocognitive reserve and brain health. Here we discuss how these results corroborate our previous work^34–36^ and that of others,^37^ to provide further experimental evidence in support of the hypothesis that EEs strengthen right hemisphere fronto-parietal networks to facilitate the phenomenon of cognitive reserve.^19,20^

The findings presented here indicate that microstructure of the right SLF1 might be specifically sensitive to the beneficial effects of environmental enrichment on neurocognitive health in older adults. Brain matter volume, indicative of age-related atrophy, is often used as a proxy of ageing brain health.^6,9,31–33^ Previous theoretical and experimental work suggests that lifelong exposure to cognitive and social enrichment, providing the so-called cognitive reserve, can promote healthy cognitive function despite objective markers of vulnerability in the brain including atrophy^33^ or disease-related neuropathological changes (e.g. Alzheimer’s)^5^. Yet the mechanisms by which this reserve is facilitated have remained unclear.^6,7,97^ Here we observed that the extent to which neurite (axonal) dispersion is preserved within the right SLF1 is directly associated with both structural brain health (GMVa and WMVa) and cognitive function (intrinsic alertness on the ANT) in later years. More specifically, causal mediation models indicated that the associations between EE with both structural brain health and alertness were dependent on neurite dispersion of the right SLF1. This suggests that the benefits of EE to neurocognitive health in older adults depend, at least in part on the degree to which WM microstructural architecture within the right SLF1 has been altered. Future work should explore whether the beneficial effects of EE are specific to the right SLF (right fronto-parietal networks) or whether other networks also mediate the neurocognitive benefits of EE. It will be additionally important to identify whether the right SLF interacts with other structural markers of brain health including those particularly sensitive to age-related cognitive deficits (e.g. hippocampal atrophy).

Novel and complex environments require high levels of alertness, which in turn optimize the processing of upcoming signals.^67^ Functionally linked with noradrenaline,^98,99^ alertness has been associated with right lateralized fronto-parietal networks and thalamic structures,^16,66,73,100–103^ with deficits in maintaining alertness reported following right but not left hemispheric strokes.^74^ Given the hypothesized relevance of noradrenaline to neurocognitive resilience^19,20^ alertness is an ideal candidate to facilitate the beneficial consequences of EE. Our results presented here suggest that repeated exposure to EE, which likely necessitates high levels of alertness, reduces axonal dispersion within right hemisphere fronto-parietal networks, to exert beneficial effects on cognitive symptoms of ageing. Although causality cannot be inferred from our cross-sectional design, it is possible that a self-perpetuating relationship between the high levels of alertness required by EEs, well-established structural networks which support the cognitive capacity of alertness (right SLF1) and intrinsic alertness capacities. This would not only be directly in line with the hypothesis that alertness, through its association with the right lateralized noradrenergic system and the right fronto-parietal networks, is a critical determinant of cognitive reserve^20^ but would also support the rationale for designing interventions to maximally modulate alertness, through modulation of the noradrenergic system, in older adults. Given the foundational role of alertness in more global aspects of attention,^104^ improving this facet of cognition may result in a cascade of benefits for the preservation of neurocognitive health in later years. Future longitudinal and intervention work should disentangle the causal directionality of these effects, underpinning the association between EE and microstructural WM changes within the SLF. This could be combined with pharmacological manipulations, non-invasive proxies of noradrenaline (e.g. pupilometry), and parallel work in animal models to assess whether the degree to which WM fronto-parietal plasticity is induced depends on noradrenergic action. Finally, it would be important to explore whether pre-existing individual differences in alertness and/or WM structure of the SLF influence the extent to which enrichment impacts the WM (i.e. the ‘chicken or egg’ question of cognitive reserve).

The SLF is a WM fronto-parietal association pathway that consists of three branches.^38^ The most dorsal branch (the SLF1) has projections to the frontal eye fields, premotor cortex and the intralparietal sulcus^18^. These regions comprise a functional network often referred to as the dorsal attention or dorsal fronto-parietal network.^100,105–107^ This network is activated by internal goals and expectations and links the processing of sensory information, including the formation of perceptual decisions with relevant motor commands.^42,106^ As such, it is likely that WM architecture within the dorsal SLF facilitates the coordination and communication of information between sensory, decision and motor regions to subsequently influence the efficiency of an individual’s response.^42^ Our findings may suggest that a lifetime of intrinsic alertness ‘training’, afforded by high EE, may induce WM plasticity within the right SLF1 to facilitate better intrinsic alertness in older years. In our cohort, these effects were driven by the complexity of professional and occupational engagements. Future work in the larger population-level cohort and longitudinal ‘big data’ studies should investigate the optimal ways to induce WM plasticity within the right SLF1. In addition, future work should assess whether in vulnerable cohorts novel intervention techniques (e.g. brain stimulation and neurofeedback) could be used to induce similar changes to the structural organization within this pathway. Addressing these hypotheses would provide increasing support for the utility of the right SLF as a marker of resilience in older adults and facilitate translational avenues to optimize interventions aimed at preventing age-related cognitive decline.

The results presented in this manuscript raise the intriguing possibility that WM plasticity within the right fronto-parietal networks may be induced by EEs to facilitate resilience to cognitive decline later in life. Here we show that EE was associated with decreased dispersion of axons (lower ODI) specifically within the right SLF. Previous studies contrasting younger and older participants have indicated that increased dispersion of axons is one of the indicators of age-related brain changes indicative of poorer cognitive function.^55,57,58,108^ Moreover, previous reports assessing the effect of age on WM have demonstrated that NODDI-derived measures outperform measures derived from DTI.^55^ In agreement with Kodiweera *et al*., our data similarly point to greater sensitivity of ODI compared with DTI-derived FA, such that our associations between the SLF and behaviour were observed specifically for ODI and not FA (although we found weak bayesian support for an association between FA of the right SLF2 with EE). Our findings are in line with growing evidence from both rodent and human studies that experience and training induce WM plasticity, reflected through changes in WM microstructural properties in the adult brain.^29,109,110^ One of the proposed mechanisms underlying this WM plasticity is activity-dependent changes in myelination (for review see Sampaio-Baptista *et al.*^111^). Myelination and the NODDI-derived parameters used here are somewhat related but predominantly complementary measures of WM microstructure.^57,112^ Thus, future investigations with both animal and human participants should disentangle the precise biological basis underpinning the impact of a lifetime of EE on neurocognitive health. As one of the limitations of the present study is cross-sectional design, any future work should also aim to gather evidence from longitudinal studies directly exploring any relationships. It should be also noted here that despite improved estimations of microstructural features of the WM with NODDI compared with DTI, ODI (axonal dispersion) measures, which estimate the degree of fibre coherence, could be still affected by crossing with other WM tracts. As such, it is possible that our findings could be driven, at least to some extent by changes to other right fronto-parietal pathways which cross the SLF1.

One caveat to our findings is that factors which may have drawn individuals to high levels of EE throughout their lifetime (including socio-economic demographics, personality, genetic and early-life cognitive abilities) were not available for investigation. As such we cannot rule out the possibility that alternative mediating factors are contributing to our observed associations between environment, the brain, and behaviour. However, several points suggest this was not the case. First, previous work with monozygotic (identical) twin studies has shown EE positively impacts cognition over and above genetic factors.^113^ Moreover, our effects of EE were driven by the professional and not education subscale of the CRIq, suggesting that early-life cognitive engagement was not the critical influence in this cohort. Finally, increasing evidence suggests that the ageing brain maintains sufficient plasticity such that it can be targeted in later years to increase reserve and resilience.^114–116^ Thus, further work providing insights into multiple factors accounting for the diverse cognitive ageing trajectories and examining the complex interplay between the different facets of EE throughout the lifespan is required to design the best possible and perhaps personalized interventions mitigating or preventing age-related cognitive decline.

Estimating exposure to EEs is challenging for several reasons. For example, it is possible that on an individual level, different factors (proxy measures, or the degree of *engagement* with, as opposed to *exposure* to, a given environment)^117^ will impact the extent to which EE positively benefits neurocognitive health. In other words, perhaps the type of environmental enrichment is less important than the level of cognitive engagement and alertness demanded which determines the extent to which the right hemisphere SLF is strengthened. In addition, different proxy measures have different lifetime dimensions and timescales of impact e.g. childhood versus early adulthood versus later years.^118^ We propose that the complex issue of how best to increase brain resilience is best addressed through large longitudinal studies incorporating big data, activity tracking and experiential sampling. Here, we provide an objective marker in the brain that can be trialled in these larger trials as a candidate pathway supporting cognitive resilience in later years.

Although the beneficial effects of EE for neurocognitive resilience are well established, the mechanisms by which this occurs are still under considerable debate. The findings presented here, in concert with our previous findings,^34,36^ suggest that anatomical properties within the right fronto-parietal networks might be neural correlates of resilience in the ageing brain. In further support of this hypothesis, brain stimulation targeting the right fronto-parietal networks not only improves behavioural and EEG markers of attention but also temporarily alters the lateralized impact of lifetime experiences in low reserve individuals such that it resembles that of their high reserve peers.^34,35^ Although neuropathological markers of Alzheimer’s disease are associated with cognitive deficits, over 50% of the variability in symptomatology still remains unexplained.^119,120^ We propose that WM properties of the right FPN be tested as objective markers of resilient neurocognitive ageing to help capture the heterogeneity associated with the clinical presentation of Alzheimer’s disease and be used to assay the efficacy of interventions aimed at ameliorating age-related cognitive decline.

## Supplementary Material

fcac080_Supplementary_Data

## Abbreviations

ANT =: Attention Network Test
CRI =: Cognitive Reserve Index
CRIq =: Cognitive Reserve Index questionnaire
dMRI =: diffusion MRI
DTI =: diffusion tensor imaging
EE =: enriched environment
FA =: fractional anisotropy
FP =: first fixation period
FPN =: fronto-parietal network
GM =: grey matter
GMVa =: grey matter volume atrophy
ICVF =: intra-cellular volume fraction
LC-NE =: locus coeruleus norepinephrine
NODDI =: neurite orientation dispersion and density imaging
ODI =: orientation dispersion index
rSLF =: right superior longitudinal fasciculus
RT =: reaction time
SLF =: superior longitudinal fasciculus
WM =: white matter
WMVa =: white matter volume atrophy
