# Supplementary material for: Right fronto-parietal networks mediate the neurocognitive benefits of enriched environments
Source: Brain Commun. 2022 Mar 28;4(2):fcac080. doi: 10.1093/braincomms/fcac080 (PMC9035529; doi:10.1093/braincomms/fcac080)
Supplement: fcac080_Supplementary_Data [file Original Submission - Decision Letter.pdf › Original Submission - Decision Letter.pdf]

Original Submission – Decision Letter

24-Oct-2021

Dear Dr. Chechlacz,

Thank you for your recent submission BRAINCOM-2021-308
entitled "Right fronto-parietal networks mediate the neurocognitive benefits of enriched environments" to Brain Communications.

Your manuscript has been reviewed, and while the reviewers had largely positive comments, they have some constructive critiques that I think you could address in a revision of the manuscript. Therefore, I invite you to respond to the reviewers' comments and revise your manuscript. The reviewers' comments are included at the bottom of this message.

In addition to the reviewers' comments, please also address our Scientific Editor's comments added below to comply with our requirements for presentation of scientific data at Brain Communications.

Please format your revised manuscript according to our Instructions to Authors https://academic.oup.com/braincomms/pages/General_Instructions – a summary of key points is attached to help with this.

To revise your manuscript, click the link below or log into https://mc.manuscriptcentral.com/braincom and enter your Author Centre, where you will find your manuscript title listed under "Manuscripts with Decisions." Under "Actions," click on "Create a Revision." Your manuscript number will be appended to denote a revision.

*** PLEASE NOTE: This is a two-step process. After clicking on the link, you will be directed to a webpage to confirm. ***

https://mc.manuscriptcentral.com/braincom?URL_MASK=0c58514ae9e84ff7ad4591e28b5b72d6

Please upload a revised manuscript with the changes you have made shown either in coloured text or using "track changes".

When submitting your revised manuscript, you will be asked to respond to the reviewer(s) comments. Please be as specific as possible in your response to all of the comments.

The editorial team at Brain Communications also aims to increase transparency in scientific publishing and to demonstrate the value that peer review brings to the scientific process. Please therefore consider whether you are willing to have reviewer comments on your paper and your response published as supplementary data with the paper, if it is accepted. This is entirely optional and will only be done if both the authors and reviewers agree. When submitting your revision you will have the opportunity to update the answer you entered previously.

IMPORTANT: Your original files are available to you when you upload your revised manuscript. Please delete any redundant files before completing the submission.

Because we are trying to facilitate timely publication of manuscripts submitted to Brain Communications, we would appreciate your revised manuscript as soon as possible. If it is not possible for you to resubmit within 3 months, the option to submit a revision will expire, and we would then consider your revised paper as a new submission. However, if you need a little more time, please get in touch and we can extend this deadline for you.

Once again, thank you for submitting your manuscript to Brain Communications and I look forward to receiving your revision.

Best wishes

Professor Tara Spires-Jones
Founding Editor, Brain Communications

Reviewer(s)' Comments to Author:

Reviewer: 1 (Helena Gellersen)

Comments to the Author
This is an interesting investigation of the neural correlates of cognitive reserve. I appreciate the hypothesis-driven approach to this brain-behaviour relationship, which was well motivated and nicely fit into a theoretical framework of underlying mechanisms of cognitive reserve. I enjoyed reading the manuscript and hope to see it published in Brain Communications.

Reviewer: 2 (An Vo)

Comments to the Author
This study used diffusion magnetic resonance imaging (dMRI) with the neurite orientation dispersion and density imaging model (NODDI) to exam the relationship between enriched environments (EE), microstructural properties of fronto-parietal white matter association pathways, grey and white matter atrophy, and cognitive functions in a group of 50 older adults. It was hypothesized that enriched environments might strengthen the right fronto-parietal networks to facilitate the cognitive processes.

The paper is interesting and well written. The authors used an up-to-date fitting method for dMRI to derive intra-cellular volume fraction and orientation dispersion index parameters which measure neurite packing density and dispersion of neurites/axons. These parameters may be more sensitive to changes of microstructural properties than fractional anisotropy measures derived from conventional diffusion tensor imaging model. Results showed that EE is associated with a lower orientation dispersion index within the right superior longitudinal fasciculus which mediates the relationship between EE, cognitive function, and grey and white matter atrophy.

This paper is based on hypothesis of fronto-parietal white matter association tracts or region (tract) of interest approach. It would be more interesting to learn from NODDI-derived parameters if there are any other networks that mediate the neurocognitive benefits of enriched environments.

While a lower orientation dispersion index within the right superior longitudinal fasciculus is associated with a better cognitive function, the mechanisms of the preservation of neurocognitive health in later years in EE people are not very clear.

Some typos were found:
Page 9, line 6-7: TE=81.5. Please provide unit for TE
Page 9, line 26: T1-weithed
Page 13, lines 38-39: R2change=02
Figure 5: …(ANT)+1

Reviewer: 3 (Seraphina Solders)

Comments to the Author
The present article seeks to improve our understanding of the neurobiological basis by which exposure to enriched environments cultivates cognitive reserve. To do this, the authors investigate the relationship between exposure to enriched environments throughout life and microstructural properties of the superior longitudinal fasciculus (measured using NODDI), grey matter atrophy, and attention in a sample of older adults. They demonstrate that exposure to enriched environments, particularly through one's occupation, is associated with a reduced orientation dispersion index within the right SLF1, which in turn mitigates grey and white matter atrophy and declines in alertness. The paper is well written, the methods are sound, and the presentation of results and general structure is clear. Further, I appreciate the discussion of methodological limitations and caveats by the authors, who interpret the results appropriately. I have mostly only minor comments about adding more details which I hope can help improve the clarity of the paper.

Major point 1: Some more details on MRI processing would be useful. In particular… Were field maps collected for susceptibility-induced distortion correction? Your MRI data acquisition section states that 5 additional b0 volumes were acquired for this purpose, so this is unclear. Additionally, was any quality assessment done of MR images? For example, were the raw images visually inspected? Were any motion measures extracted and examined from the eddy output? Finally, what tools were used for transformations (FNIRT/FLIRT?)?

Minor point 1: Page 9, lines 26-27 - there is a repeat of a sentence describing the T1 preprocessing that should be removed from the dMRI preprocessing section.
Minor point 2: Were the models linear regressions?
Minor point 3: In Figure 3, it would be helpful to add units where appropriate (milliseconds?)
Minor point 4: On page 13, last two lines of first results section – are these sentences in the right place? It is mentioned that 3 subjects were removed as outliers from the MR analysis in a section that has

nothing to do with the MR images. These sentences may be more helpful to put in earlier when you mention the removal of outliers, or perhaps the beginning of the next section which begins to discuss the MR analyses.
Minor point 5: In Figure 4, it may ease readability to indicate which relationships were significant either on the scatterplots or in the caption

Editorial Office Comments:

-Please supply a graphical abstract. A graphical abstract is a visual representation of the central finding or methodology of the paper. It will be displayed in the table of contents. Authors should strive to make the graphical abstract informative, interesting, visually appealing, and straightforward (no caption needed, no abbreviations except gene names). Please upload as a separate high resolution image file (tiff preferred). The technical requirements for the graphical abstracts are:
--size of the submitted image: 1200 pixels square at 300dpi
--font of the text: Arial 12-16 point (smaller fonts will not be legible online)

-Please add an Abbreviated Summary (up to 50 words) of your paper that captures the main purpose and conclusions of the work. The summary should state the main results written in the third person ('[1st author surname] et al. report that …') and conclusion. This summary will be used in the contents list online and should not contain any abbreviations other than those on the accepted abbreviations list and accepted gene/protein names, making it accessible to the non-specialist. Please upload it as a Word document. Allowed abbreviations: AIDS; ANOVA; AMPA; ATP; A,T,C,G; CNS; CSF; CT; DNA; ECG; EEG; EMG; GABA; HIV; MRI; NMDA; PET; PCR; RNA.

- To comply with our ethical standards, the appropriate checklist must be included (see Reporting Guideline section within 'Instructions to authors').

-Please ensure all figure legends contain appropriate N numbers and statistical tests used, along with their p-values.
