# Supplementary material for: Right fronto-parietal networks mediate the neurocognitive benefits of enriched environments
Source: Brain Commun. 2022 Mar 28;4(2):fcac080. doi: 10.1093/braincomms/fcac080 (PMC9035529; doi:10.1093/braincomms/fcac080)

POINT-BY-POINT RESPONSES TO THE REVIEWERS:
We would like to thank the Reviewers for their detailed assessment of our work. We are delighted to see that overall, all Reviewers found our work interesting and relevant. We are grateful for their constructive comments and helpful suggestions. We have now revised the manuscript, and our detailed responses to issues raised by the Reviewers are listed below. All changes in the manuscript are indicated by red text, and are systematically described below.

REVIEWER 1
Comments to the Author:
This is an interesting investigation of the neural correlates of cognitive reserve. I appreciate the hypothesis-driven approach to this brain-behaviour relationship, which was well motivated and nicely fit into a theoretical framework of underlying mechanisms of cognitive reserve. I enjoyed reading the manuscript and hope to see it published in Brain Communications."

Response:
We thank the Reviewer for their very encouraging comments and recognition of our efforts to provide evidence in support of proposed by Robertson theoretical framework of cognitive reserve.

REVIEWER 2
Comments to the Author:
This study used diffusion magnetic resonance imaging (dMRI) with the neurite orientation dispersion and density imaging model (NODDI) to exam the relationship between enriched environments (EE), microstructural properties of fronto-parietal white matter association pathways, grey and white matter atrophy, and cognitive functions in a group of 50 older adults. It was hypothesized that enriched environments might strengthen the right fronto-parietal networks to facilitate the cognitive processes.
The paper is interesting and well written. The authors used an up-to-date fitting method for dMRI to derive intra-cellular volume fraction and orientation dispersion index parameters which measure neurite packing density and dispersion of neurites/axons. These parameters may be more sensitive to changes of microstructural properties than fractional anisotropy measures derived from conventional diffusion tensor imaging model. Results showed that EE is associated with a lower orientation dispersion index within the right superior longitudinal fasciculus which mediates the relationship between EE, cognitive function, and grey and white matter atrophy."

Response:
We thank the Reviewer for their encouraging assessment of our work and helpful comments on how to improve data presentation and interpretation of findings. Detailed responses to specific suggestions are provided below.

Major points:
1. *This paper is based on hypothesis of fronto-parietal white matter association tracts or region (tract) of interest approach. It would be more interesting to learn from NODDI-derived parameters if there are any other networks that mediate the neurocognitive benefits of enriched environments.*

Response: This is an interesting avenue of work which we ourselves are keen to understand. The research carried out for this manuscript was driven by a highly specific hypothesis based on our previous work (Brosnan et al., 2017, *Cerebral Cortex*; Shalev et al., 2020, *Cerebral Cortex Communications*). More specifically, in a recent whole-brain 'blinded' analysis of grey-matter in an older adult cohort, we found that EE was specifically associated with less grey matter atrophy in a right hemisphere fronto-parietal network (Shalev et al., 2020). This added to growing evidence (e.g., Brosnan et al., 2017) that the right fronto-parietal support neurocognitive resilience in older adults (Robertson, 2013, 2014).

Following from these results, the current study was specifically motivated to explore whether white matter plasticity within these fronto-parietal networks (via the SLF) was additionally altered through exposure to enriched environments. While we had strong hypotheses about the results, we note that in the current study, we explore all 6 branches of the SLF across the two hemispheres and observe specific associations between EE and axonal dispersion of the right, but not left, hemisphere, consistent with our work and that of others (Brosnan et al., 2017; Shalev et al., 2020; Van Loenhoud et al 2017 *Human Brain Mapping*; Roberstson 2013,

2014, *Neurobiology of Aging*). To explore other networks/other white matter pathways, we would need to change the design of the current study and significantly expand all analyses which would require a significantly larger study sample, given the large number of tracts to be explored. We have started to explore these larger questions in a population-level data sets (e.g., the UK Biobank). This will provide more power to investigate this interesting and important question. As such we hope you agree that while this is an exciting avenue of future work is beyond the scope of our current investigations.

We have raised this important issue in our discussion section as an avenue of pressing interest for future work.

Updated Discussion Section

Future work should explore whether the beneficial effects of EE are specific to the right SLF (right fronto-parietal networks) or whether other networks also mediate the neurocognitive benefits of EE. It will be additionally important to identify whether the right SLF interacts with other structural markers of brain health including those particularly sensitive to age-related cognitive deficits (e.g., hippocampal atrophy).

2. *While a lower orientation dispersion index within the right superior longitudinal fasciculus is associated with a better cognitive function, the mechanisms of the preservation of neurocognitive health in later years in EE people are not very clear.*

Response: We thank the Reviewer for raising this helpful point. We fully agree with the Reviewer that the putative mechanisms of the preservation of neurocognitive health in later years in EE people were not very clear in the previous version of our manuscript. In our updated discussion we highlight that our findings indicate that microstructure of the right SLF1 might be sensitive to the beneficial effects of environmental enrichment to neurocognitive health, both in terms of cognitive function and brain atrophy as assessed by behavioural performance and brain volumetric measures, respectively. While in the discussion we propose that a potential mechanism underlying these beneficial effects is likely repeated engagement of the noradrenergic system and corresponding right fronto-parietal networks due to the high levels of alertness likely necessitated by EE, we also discuss constrains of the current study and provide suggestion for future work.

Revised Discussion Section

Novel and complex environments require high levels of alertness, which in turn optimise the processing of upcoming signals (Petersen & Posner, 2012). Functionally linked with noradrenaline (Aston Jones & Cohen 2005; Marrocco & Davidson 1998), alertness has been associated with right-lateralised fronto-parietal networks and thalamic structures (Posner & Petersen, 1990; Corbetta & Shulman, 2002; 2011; Strum & Willmes 2001; Singh-Curry & Husain, 2009; Fan et al 2005; see also Coull et al., 2000; Fan et al 2005), with deficits in maintaining alertness reported following right but not left hemispheric strokes (Posner et al., 1987; Posner, 2008). Given the hypothesised relevance of noradrenaline to neurocognitive resilience (Robertson, 2013, 2014) alertness is an ideal candidate to facilitate the beneficial consequences of EE. Our results presented here suggest that repeated exposure to EE, which likely necessitates high levels of alertness, reduces axonal dispersion within right hemisphere fronto-parietal networks, to exert beneficial effects on cognitive symptoms of ageing. Although causality cannot be inferred from our cross-sectional design, it is possible that a self-perpetuating relationship between the high levels of alertness required by enriched environments, well-established structural networks which support the cognitive capacity of alertness (right SLF1), and intrinsic alertness capacities. This would not only be directly in line with the hypothesis that alertness, through its association with the right lateralised noradrenergic system and the right fronto-parietal networks, is a critical determinant of cognitive reserve (Robertson, 2014) but would also support the rationale for designing interventions to maximally modulate alertness, through modulation of the noradrenergic system, in older adults. Given the foundational role of alertness in more global aspects of attention (Raz & Buhle, 2006), improving this facet of cognition may result in a cascade of benefits for the preservation of neurocognitive health in later years. Future longitudinal and intervention work should disentangle the causal directionality of these effects, underpinning the association between EE and microstructural white matter changes within the SLF. This could be combined with pharmacological manipulations, non-invasive proxies of noradrenaline (e.g. pupilometry), and parallel work in animal models to assess whether the degree to which white matter fronto-parietal plasticity is induced depends on noradrenergic action. Finally, it would be important to explore whether pre-existing individual differences in alertness and/or white matter

structure of the SLF influence the extent to which enrichment impacts the white matter (i.e., the 'chicken or egg' question of cognitive reserve).

Minor points:

Some typos were found:
Page 9, line 6-7: TE=81.5. Please provide unit for TE
Page 9, line 26: T1-weithed
Page 13, lines 38-39: R2change=02
Figure 5: …(ANT)+1"

Response: All these typos have been corrected in the revised manuscript.

Reviewer 3

Comments to the Author:

The present article seeks to improve our understanding of the neurobiological basis by which exposure to enriched environments cultivates cognitive reserve. To do this, the authors investigate the relationship between exposure to enriched environments throughout life and microstructural properties of the superior longitudinal fasciculus (measured using NODDI), grey matter atrophy, and attention in a sample of older adults. They demonstrate that exposure to enriched environments, particularly through one's occupation, is associated with a reduced orientation dispersion index within the right SLF1, which in turn mitigates grey and white matter atrophy and declines in alertness. The paper is well written, the methods are sound, and the presentation of results and general structure is clear. Further, I appreciate the discussion of methodological limitations and caveats by the authors, who interpret the results appropriately. I have mostly only minor comments about adding more details which I hope can help improve the clarity of the paper."

Response:

We thank the Reviewer for the insightful and encouraging assessment of our work including number of helpful suggestion to improve clarity of methods and presentation of results.

Major points:

Some more details on MRI processing would be useful.

1. Were field maps collected for susceptibility-induced distortion correction? Your MRI data acquisition section states that 5 additional b0 volumes were acquired for this purpose, so this is unclear.

Response: We agree with the Reviewer's suggestion to include more detailed information about MRI data pre-processing. In the previous manuscript version we have only referenced technical papers detailing pre-processing steps and indeed, as a result, basic key information has been missed. In the revised manuscript, we have added this information. We thank the Reviewer for the helpful suggestion to clarify the dMRI pre-processing steps, specifically the susceptibility-induced distortion correction. We are sorry for the confusing statement, that was a typo. We used 1 blip-reversed b=0 image to correct for susceptibility distortions in accordance with Andersson et al., 2003. This has been corrected in text.

2. Additionally, was any quality assessment done of MR images? For example, were the raw images visually inspected? Were any motion measures extracted and examined from the eddy output?

Response: Following Reviewer's suggestion, we have added information about quality assessment. "All scans were visually inspected during and after the acquisition to ensure that there have not been any artefacts associated with excessive head movement and signal dropouts. All data passed this quality assessment."

3. *Finally, what tools were used for transformations (FNIRT/FLIRT?)?*

Response: In case of T1 pre-processing a non-linear registration to MNI152 space using FNIRT was applied. UK biobank pipeline (Alfaro-Almagro et al., 2018) was applied to the diffusion data. Specifically, first the corrected dMRI data were linearly registered to structural space, using a rigid body transformation (using FLIRT). Structural data were non-linearly registered to standard space (using FNIRT). Concatenating the two provided

a non-linear transformation from diffusion to standard space. This information including relevant references has been added to the methods section.

Minor points:

1) *Minor point 1: Page 9, lines 26-27 – there is a repeat of a sentence describing the T1 preprocessing that should be removed from the dMRI preprocessing section.*

Response: We have removed this sentence as indeed it was repeated by mistake.

2) *Minor point 2: Were the models linear regressions?*

Response: Indeed, we employed stepwise linear regression analyses. The procedure for this has been clarified in the manuscript.

3) *Minor point 3: In Figure 3, it would be helpful to add units where appropriate (milliseconds?)*

Response: We have amended Figure 3 and specified these units as milliseconds (ms), or delta ms (Δ ms) where appropriate.

4) *Minor point 4: On page 13, last two lines of first results section – are these sentences in the right place? It is mentioned that 3 subjects were removed as outliers from the MR analysis in a section that has nothing to do with the MR images. These sentences may be more helpful to put in earlier when you mention the removal of outliers, or perhaps the beginning of the next section which begins to discuss the MR analyses.*

Response: We thank the Reviewer for highlighting this. Three outliers were removed in total. Two as outliers in neuroimaging measures, which were defined as any scores greater or less than three times the inter-quartile range (IQR). Note that both participants' values fell within the acceptable ranges indicating that there were no issues with the reconstructed diffusion data. One participant provided incomplete information on the Cognitive Reserve Index questionnaire and was removed from the statistical analyses as we were unable to calculate the CRIq score. We apologise for this error in the original version of the manuscript. We have revised this information, and as suggested by the Reviewer included it in the appropriate subsection of Methods detailing the outliers removal, which proceeds the Statistical Analysis subsection.

5) *"Minor point 5: In Figure 4, it may ease readability to indicate which relationships were significant either on the scatterplots or in the caption"*

Response: We have amended the Figure 4 caption accordingly.

Reviewer 4

Summary

In their manuscript, Brosnan and colleagues investigate neural correlates of enriched environments in healthy older adults using structural neuroimaging methods. Enriched environments are suggested to contribute to cognitive reserve, resources which allow an individual to remain cognitively healthy and even stave off some of the negative effects of ageing or dementia. The authors hypothesised that structural features of the right lateralised fronto-parietal regions may be associated with exposure to enriched environments and may in turn be related to attentional capabilities. Using novel structural MRI methods (Neurite Orientation Dispersion and Density Imaging) and well-established measures of cognitive reserve and attention, the authors show a specific relationship between neurite density of the right superior longitudinal fasciculus (SLF), alertness and exposure to enriched environments by virtue of higher occupational complexity. These relationships were not present for other structural measures such as fractional anisotropy. Interestingly, lower neurite dispersion in SLF1 mediated the relationship between environmental enrichment and alertness as well as the association between enrichment and whole-brain white and grey matter. These findings contribute to our understanding of cognitive reserve and are a valuable addition to the current literature. I recommend publication of this manuscript in Brain Communications provided the authors can address the following comments.

Response:
We thank the Reviewer for the encouraging evaluation of our research and the novel contribution in support of the theoretical framework of cognitive reserve. We appreciate the constructive comments and suggestions which we believe have strengthened the evidence supporting our findings. We have addressed these points in detailed response below.

Major comments:

1. The authors mention a stepwise approach for model building. Please specify whether this was done using an automated method (e.g. order was selected on some prespecified criterion) or whether predictors were added based on an a priori basis (e.g. hypothesis driven).

Response: Although we had a strong hypothesis from our previous work (Shalev, Brosnan, & Chechlacz 2020, Cerebral Cortex Communications; Brosnan et al. 2017 Cerebral Cortex) that enriched environments would specifically impact the right hemisphere, we entered all six SLF branches (SLF 1-3, left and right hemisphere) to the stepwise model in a 'blinded' fashion. This approach essentially runs multiple linear regression analyses several times, removing the weakest variable at each iteration. At the end of this modelling procedure (which is fully automated with no involvement from the researcher), you are left with a (set of) variable(s) which best capture(s) variance in the dependent variable (or no variable in the case where no variance is explained).

2. Please also provide statistics regarding model fit improvement rather than relying on statistical significance of the F-test in model selection. For instance, when deciding on exclusion of other SLF branches, decisions should be based on the use of multiple fit metrics. Akaike or Bayesian Information Criterion would be ideal.

Response: We thank the Reviewer for this suggestion which has greatly improved our manuscript. We have included Bayesian Information Criterion as follow-up analyses to all frequentist statistics in text. We are happy to report that these additional analyses have instilled further confidence in our conclusions with regards specific influences of EE on the right SLF 1.

3. Furthermore, in order to provide more information on the meaningfulness of the MRI, the CRIq, and the ANT predictors, respectively, using an effect size such as Cohen's f 2 to accompany the F-statistic for model comparisons would be recommended. The large number of statistical tests carried out calls for an approach that is not entirely based on p-values and additionally considers the magnitude of the effects and the confidence in the model estimates. These should in turn be discussed to provide the reader with an understanding of the importance of the effect.

Response: All significant statistical tests have been supplemented with Cohen's F^2 measures of effect sizes and confidence intervals. This was calculated as R2/(1 − R2). As per our response above we have added Bayesian statistics and as suggested the new results are reported and briefly discussed in the revised manuscript.

4. To bolster your claim about the specificity of the MRI-EE associations, it would be helpful to include a Bayesian analysis. For instance, you could calculate the Bayes Factor that is obtained when comparing your EE model with age only against an EE model with ICVF; then do the same for FA. This would allow you to make more meaningful claims about the null effect. As it stands, it is unclear whether sample size could have contributed to the null effect for these measures (e.g. for FA, a p-value around .13 could suggest insufficient power). The same can be done in the comparison of different CRIq subscales and ANT scores, respectively.

Response: Following Reviewer's suggestion Bayesian statistics have been added throughout, please see revised methods and results sections. While our main conclusions with regards the association between the right SLF 1 and EE remain unchanged, the Bayesian approach additionally revealed that white matter properties (ODI and FA) of the right hemisphere SLF2 also varied (albeit weakly) according to EE. These updated results are included in the revised manuscript.

5. -Please indicate for all chosen models how model diagnostics were carried out. Which assumptions were tested and were they violated (normality of residuals, heteroskedasticity, etc.)? Were outliers and influential cases identified? How? What robustness tests were carried out if assumptions were

violated or outliers/influential cases were found? How did model estimates change after problematic cases were removed? This could be described in a supplementary document.

Response: Normality of residuals and heterskedascticity were tested for all analyses by visual inspection of the residuals, and formal analysis using the Shapiro Wilks test of normality. For your convenience all plots and analyses are included at the end of this 'Response to Reviewers' document. After removal of two extreme outliers (as per the original submission), none of the data were problematic. Please see the next point for a discussion on outliers. We have included a line in the methods of the revised manuscript to outline this procedure.

Minor comments:

1) Outlier removal (pg. 10): please specify the number of participants excluded based on MRI metrics (this is done on pg. 13 but should also be noted here) and explain any potential reasons for why these participants may have been outliers (e.g. image quality issues, other technical errors); if there was no apparent reason and multiple participants were identified as outliers, these cases should not be blindly removed but rather, results should be shown with and without the case removed, or robust statistics should be used (e.g. in a supplementary analysis).

Response: We thank Reviewer 4 for raising this point and apologise that we made an error here in our reporting. We checked all variables for outliers, which were defined as any scores greater or less than three times the inter-quartile range (IQR). As per these criteria, one participant was identified as an outlier on the ICVF metric for all 6 branches of the SLF. Another participant was an outlier (less than 3 times the IQR) on 5/6 branches of the SLF. We have outlined the values for each of these outliers in the table below. Note that both participants' values fell within the acceptable ranges indicating that there were no issues with the reconstructed diffusion data. A final participant provided incomplete information on the Cognitive Reserve Index questionnaire and was removed from the statistical analyses as we were unable to calculate the CRIq score. We apologise that in the original version of the manuscript we had incorrectly written that three data points were excluded as imaging outliers. We have now corrected this in text.

	Outlier Value	Mean	Outlier Value	Mean
	**ICVF**		**ODI**	
SLF1 L	.94	.59	.15	.25
SLF1 R	.96	.58	.19	.26
SLF2 L	.91	.59	.17	.28
SLF2 R	.92	.61	.15	.28
SLF3 L	.89	.60	.16	.27
SLF3 R	.92	.60	.19	.27

While we appreciate all of Reviewer's suggestions for assessing the robust nature of the associations reported in our data and were delighted to conduct the other analyses recommended by Reviewer including Bayesian inference and normality checks, we do not believe repeating our analysis pipeline to include these two "neuroimaging outliers" would be informative. As highlighted in the table above, these are clear outliers in the data and their inclusion would violate the assumptions of normality and invalidate our statistical models.

2) Which software was used for the statistical analysis?

Response: Statistical analyses were conducted using a combination of JASP (version 0.11.1, JASP Team 2019; Wagenmakers et al., 2017), IBM SPSS Statistics, Version 27, and MATLAB r2021a. This information has been added to the revised manuscript.

3) It would be helpful to indicate that the network score in Fig 3b reflects the difference in reaction times between two conditions (e.g. by adding delta RT in brackets to the title of the y-axis).

Response: The Figure 3 has been amended accordingly.

4) It would be helpful to show the scatter plot for the correlation between the alertness network score and mean reaction times. This would allow a better interpretation of the alertness score for

individual participants (e.g. in case some participants did indeed use the cue more efficiently, we would expect them to be a multivariate outlier on this plot).

Response: Following Reviewer's suggestion the scatter plot has been added to Figure 3, please see panel D of the revised figure.

5) Table 1:
   - When only looking at this table without reading the text, it is unclear whether it demonstrates multiple separate models with neurite dispersion as dependent variable or one model with EE, Grey Matter Volume Atrophy (GMVa), White Matter Volume Atrophy (WMVa), and Alertness all in one model. Presumably it refers to multiple separate models. It would be good to clarify this either in the header or a footnote.
   - Showing the standard error and confidence for the standardised beta would be more useful than showing the unstandardised coefficient. Unstandardised coefficients are helpful to calculate the predicted scores, but for the interpretation of the uncertainty about model estimates, SEs and CIs should be shown for the standardised beta. Also note that all statistics should be in cursive.

Response: Following Reviewer's suggestions we amended Table 1 caption and all statistics are reported in cursive in the revised manuscript. Please see our response below with regards to how we report statistics throughout the revised manuscript.

6) Pg. 14, lines 33: where this is not reported in Table 1, provide standardised regression coefficients and confidence intervals for your estimates of interest in the final selected model. This also applies to the entire manuscript.

Response: We thank the Reviewer for all helpful comments about the statistical analyses and for suggesting the additional statistics to further explore the robust nature of our reported effects. We have now added the standardised beta coefficients, confidence intervals, effect sizes, and model comparisons using BIC to all of our analyses. As a result, our manuscript includes a lot of statistical information. While we appreciate the value in adding the aforementioned statistics, with regards SE and CIs, we have opted to include SE for all unstandardised beta weights, and confidence intervals for standardised beta weights. This offers our reader information about model uncertainty for both scenarios without over-cluttering our (already-lengthy) results section with information that does not add additional value. We hope, particularly in light of the additional Bayesian analyses in support of our findings, that this is an acceptable solution.

7) One interesting aspect to discuss: for individuals with less complex occupations, can leisure activity make up for less occupational complexity? This may be particularly interesting in the context of your discussion on novel interventions. This may not be possible to answer with the current sample size but is an interesting question for future directions. On that note: what was the relationship between subscales of the CRIq? If education and occupation scores do not correlate, this may be an interesting result to add to the discussion.

Response: We thank the Reviewer for this interesting suggestion. In the original paper by Nucci et al (2012), a group who developed CRIq, reported substantial correlations between composite CRIq score and the three subscales (r=0.72-0.78) and weaker correlations between the three subscales, with education and occupation showing the strongest association. This was also the case in our sample of older individuals (results included in the table below). While these results (i.e., BF_{10}<3) suggest that leisure and occupational subscales are only moderately related and somewhat orthogonal, the question of whether leisure activities can facilitate neuroprotective effects in cases where individuals have low levels of occupational (or educational) engagement remains unclear. As suggested by the reviewer, this is an important question which would facilitate the development of scalable novel interventions. Alas, within the current sample size, it is not possible to disentangle these questions. In the revised manuscript we now raise the importance of this point, and suggest that future work address these intriguing questions in larger scale population level data.

	CRI Occupation	CRI Leisure	CRI Education
CRI Occupation			
CRI Leisure	*r*=.34, *p*=.02<br><br>BF_{10}=2.41		
CRI Education	**r=.59, p<.0005**<br><br>**BF_{10}=1205.07**	*r*=.33, *p*=.02<br><br>BF_{10}=2.18	

Updated Discussion Section

Estimating exposure to enriched environments is challenging for several reasons. For example, it is possible that on individual level, different factors (proxy measures, or the degree of engagement with, as opposed to exposure to, a given environment; Park et al., 2014) will impact the extent to which EE positively benefits neurocognitive health. In other words, perhaps the type of environmental enrichment is less important than the level of cognitive engagement and alertness demanded which determines the extent to which the right hemisphere SLF is strengthen. In addition, different proxy measures have different lifetime dimensions and timescales of impact e.g., childhood versus early adulthood versus later years (Kartschmit et al., 2019). We propose that the complex issue of how best to increase brain resilience is best addressed through large longitudinal studies incorporating big data, activity tracking, and experiential sampling. Here we provide an objective marker in the brain which can be trialled in these larger trials as a candidate pathway supporting cognitive resilience in later years.

8) Finally, the authors touch upon the possibilities of other mediating factors. They should also stress that a main limitation is the cross-sectional design of this study and that longitudinal data would further be needed to support the claim regarding causality.

Response: In accordance with Reviewer suggestion we expanded discussion about other factor as well as discuss the limitations of cross-sectional design and need for longitudinal follow up.

9) Language and typos:
   - Pg 6., line 54: “flankers”, not “flakers”
   - Pg. 9, line 26: “weighted”, not “weithed”
   - Pg. 13, line 39: statistic for R2 is missing a full stop
   - Pg. 14, line 26: “variable”, not “variance”
   - Pg. 17, line 48: “hypothesis” is a more fitting term than “theory”
   - Pg. 18, line 34: should be “high levels of alertness”

Response: All these typos and omissions have been corrected in the revised manuscript.

Editorial Office Comments:

1. *Please supply a graphical abstract. A graphical abstract is a visual representation of the central finding or methodology of the paper. It will be displayed in the table of contents. Authors should strive to make the graphical abstract informative, interesting, visually appealing, and straightforward (no caption needed, no abbreviations except gene names). Please upload as a separate high resolution image file (tiff preferred). The technical requirements for the graphical abstracts are:*
--size of the submitted image: 1200 pixels square at 300dpi
--font of the text: Arial 12-16 point (smaller fonts will not be legible online)

Response: The graphical abstract has been supplied in accordance with the technical requirements.

2. -Please add an Abbreviated Summary (up to 50 words) of your paper that captures the main purpose and conclusions of the work. The summary should state the main results written in the third person

(‘[1st author surname] et al. report that …’) and conclusion. This summary will be used in the contents list online and should not contain any abbreviations other than those on the accepted abbreviations list and accepted gene/protein names, making it accessible to the non-specialist. Please upload it as a Word document. Allowed abbreviations: AIDS; ANOVA; AMPA; ATP; A,T,C,G; CNS; CSF; CT; DNA; ECG; EEG; EMG; GABA; HIV; MRI; NMDA; PET; PCR; RNA.

Response: Abbreviated summary has been included with the revised submission.

3. To comply with our ethical standards, the appropriate checklist must be included (see Reporting Guideline section within 'Instructions to authors').

Response: The manuscript has been prepared in accordance with the STROBE checklist for cross-sectional studies. A completed STROBE checklist has been included with the revised submission.

4. -Please ensure all figure legends contain appropriate N numbers and statistical tests used, along with their p-values.

Response: All figure legends have been amended accordingly.

HETEROSKEDASTICITY & NORMALITY OF RESIDUAL CHECKS

EE as a function of age & ODI of all 6 SLF branches

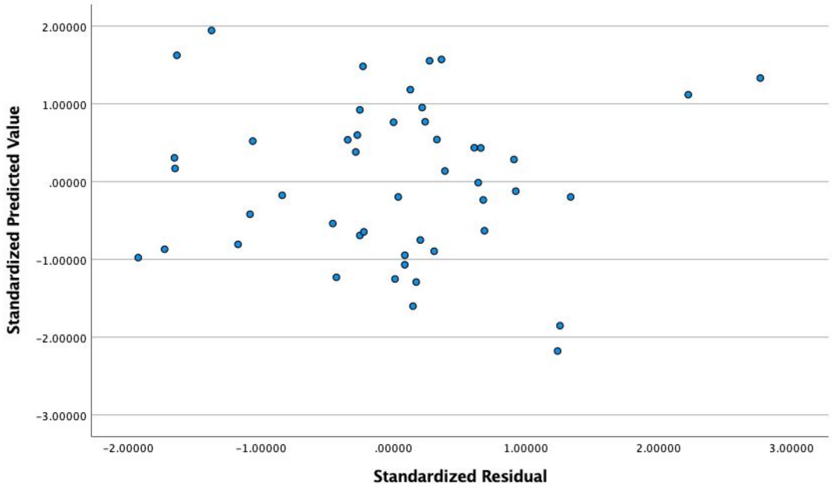

Shapiro-Wilk test of the residuals: W(47)=.96, *p*=.11.

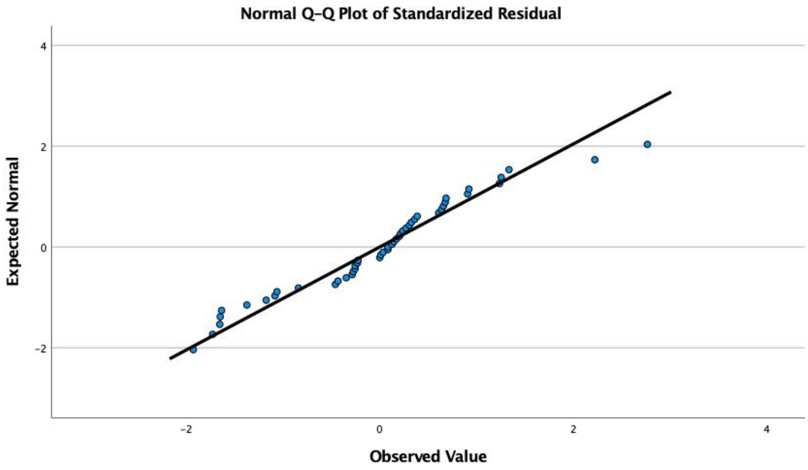

EE (CRIq composite) as a function of ODI r SLF1

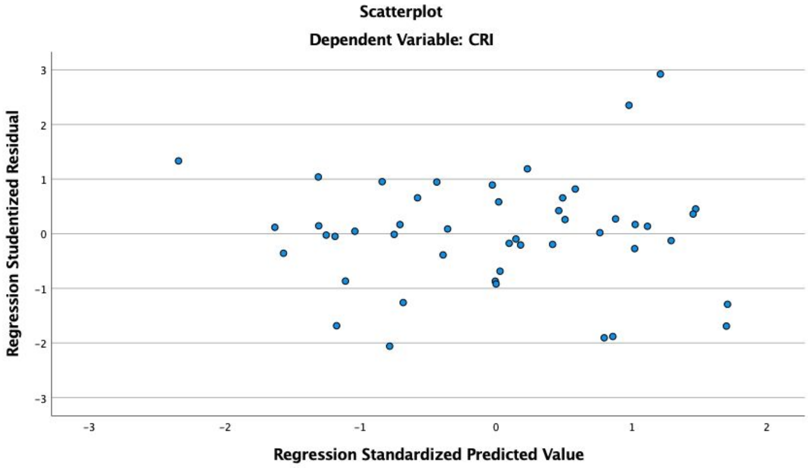

Shapiro-Wilk test of the residuals: W(47)=.96, *p*=.09.

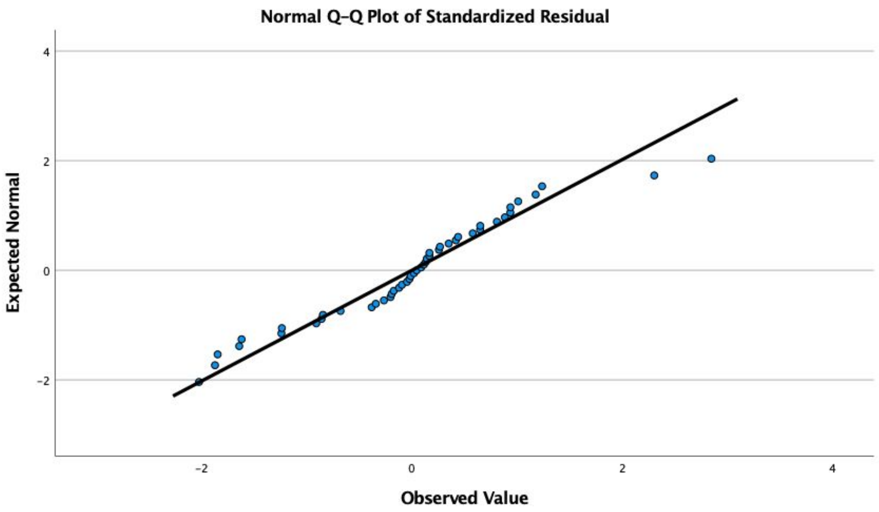

EE as a function of age & FA of all 6 SLF branches

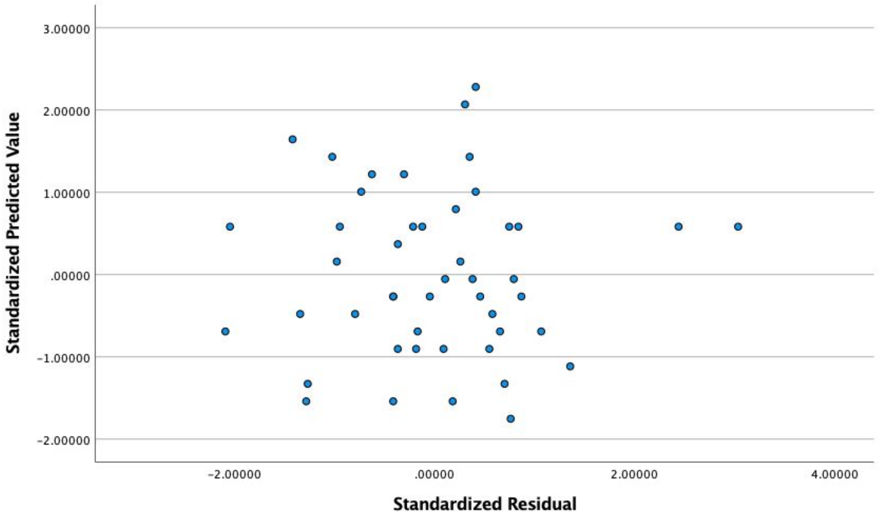

Shapiro-Wilk test of the residuals: W(47)=.96 *p*=. 11

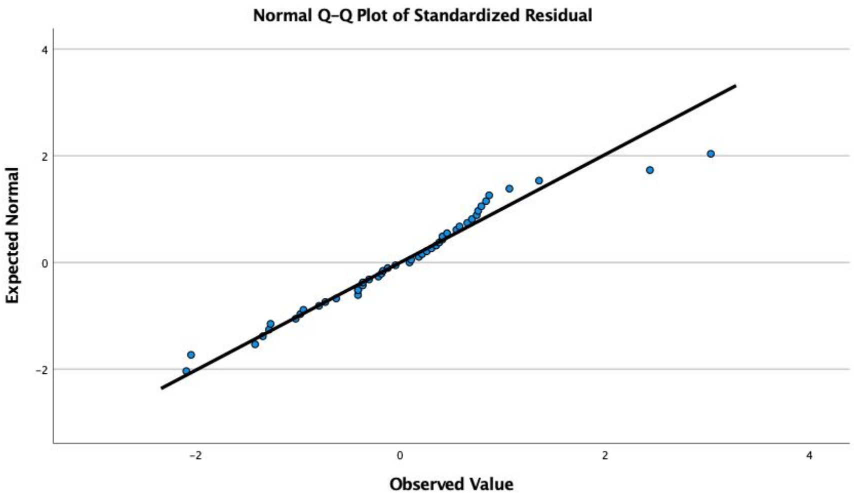

EE as a function of age & ICVF of all 6 SLF branches

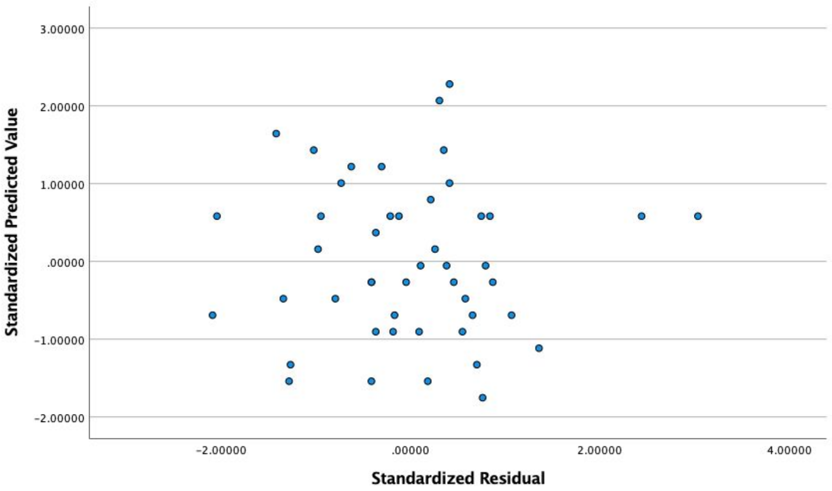

Shapiro-Wilk test of the residuals: W(47)=.96. *p*=.14

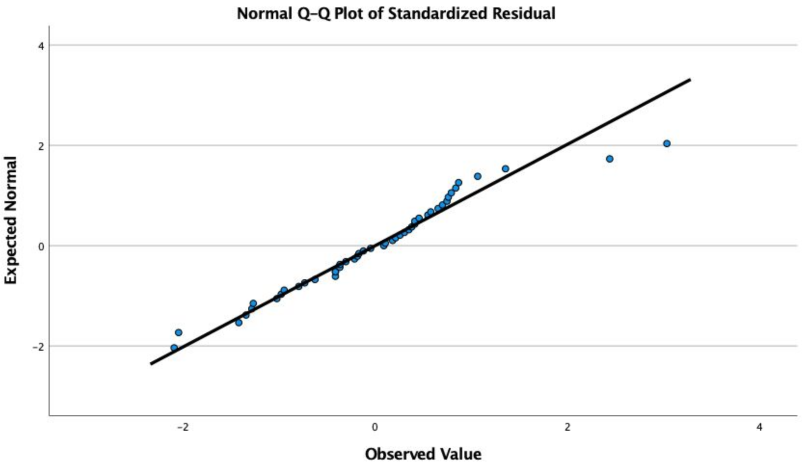

EE (work subscale) as a function of ODI r SLF1

Visuaisation of the Residuals versus predicted values

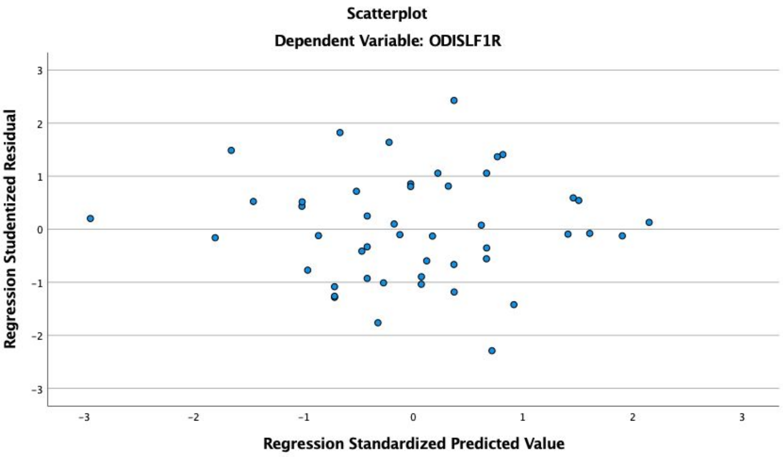

Shapiro-Wilk test of the residuals: W(47)=.99, *p*=.997.

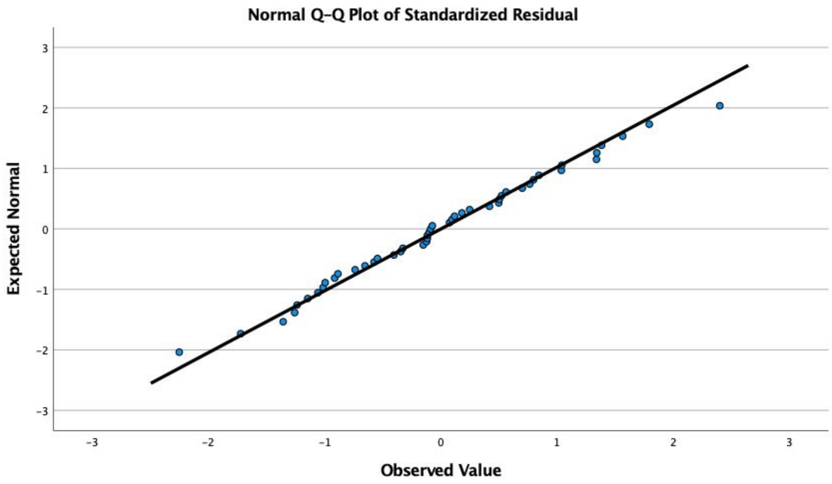

Grey Matter Volume atrophy as a function of 6 SLF branches (ODI)

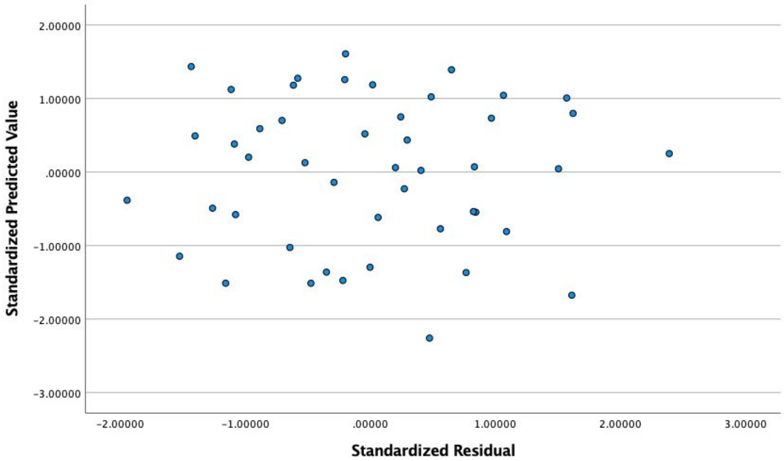

Shapiro-Wilk test of the residuals: W(47)=.99, *p*=.86

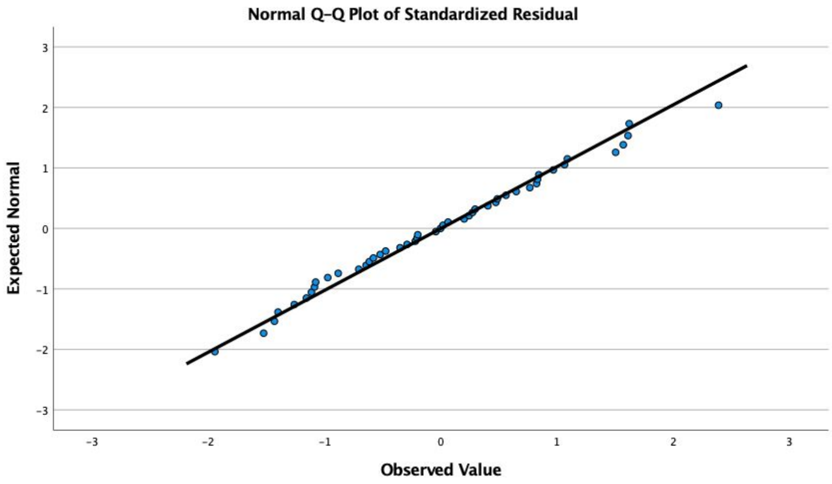

Grey Matter Volume atrophy as a function of ODI right SLF1

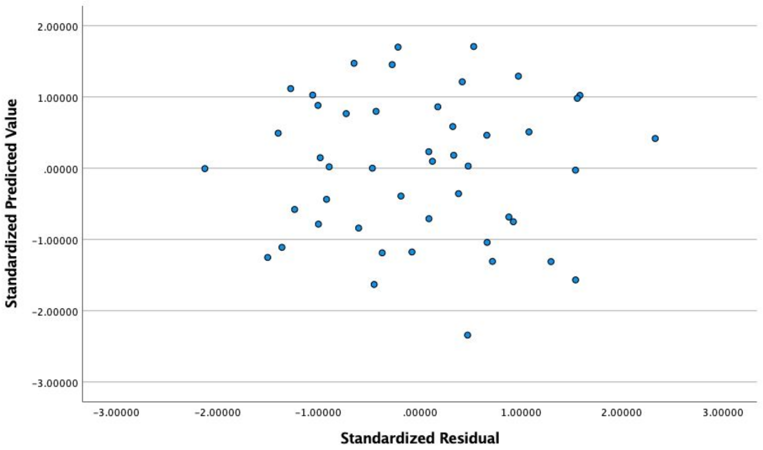

Shapiro-Wilk test of the residuals: W(47)=.99, *p*=.84

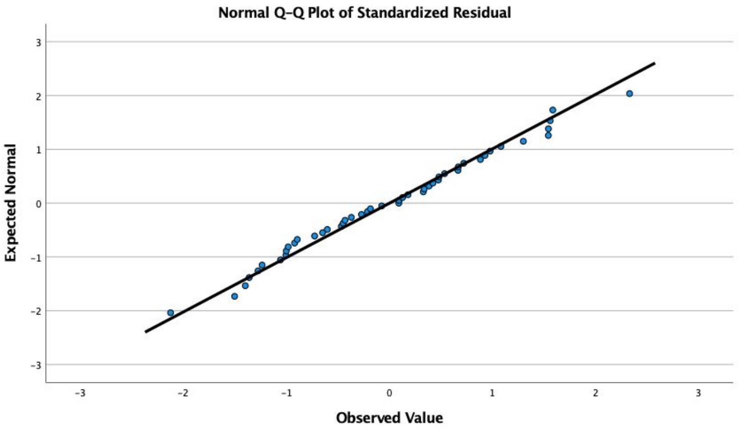

White Matter Volume atrophy as a function of 6 SLF branches (ODI)

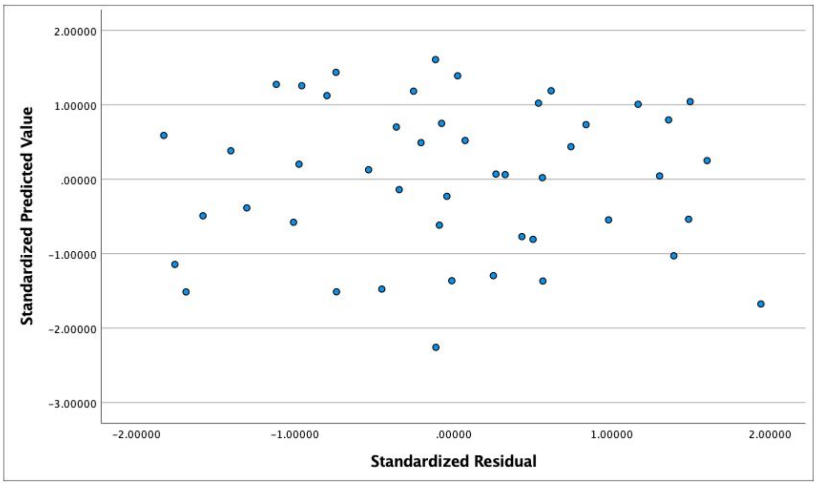

Shapiro-Wilk test of the residuals: W(47)=.98, *p*=.53

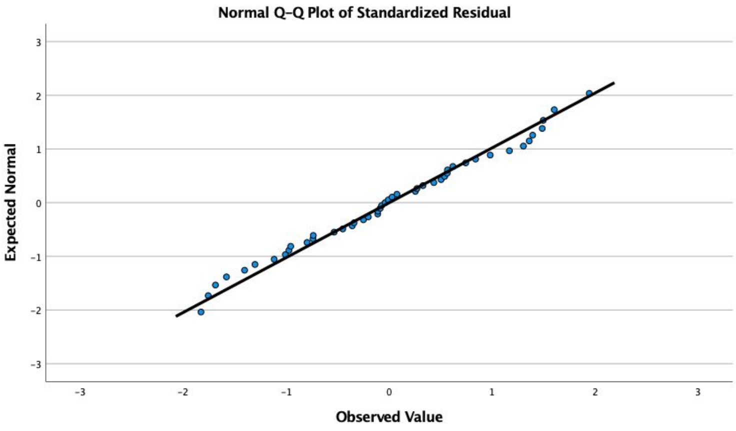

White Matter Volume atrophy as a function of ODI r SLF1

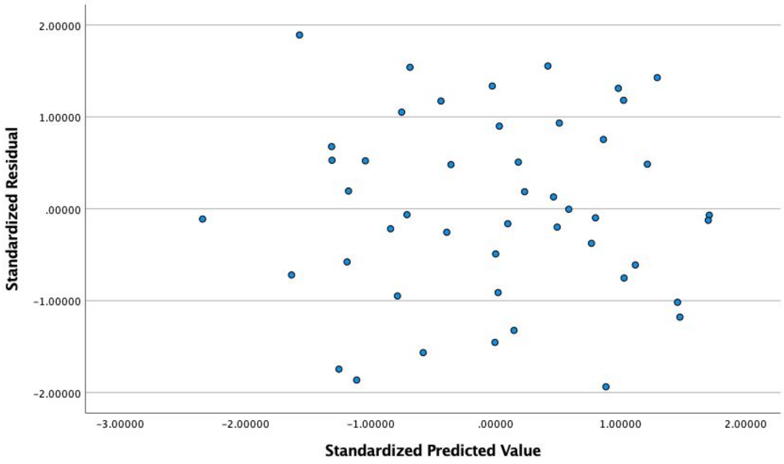

Shapiro-Wilk test of the residuals: W(47)=.98, *p*=.53

ODI rSLF1 as a function of three ANT subscales

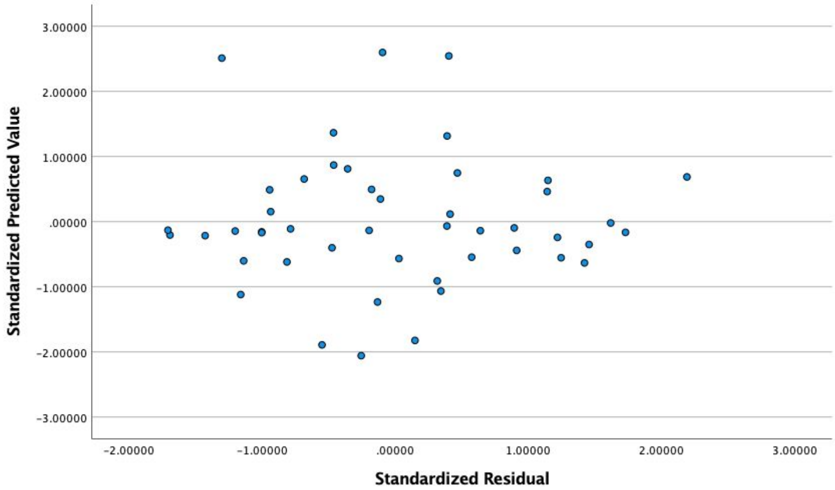

Shapiro-Wilk test of the residuals: W(47)=.98, *p*=.46

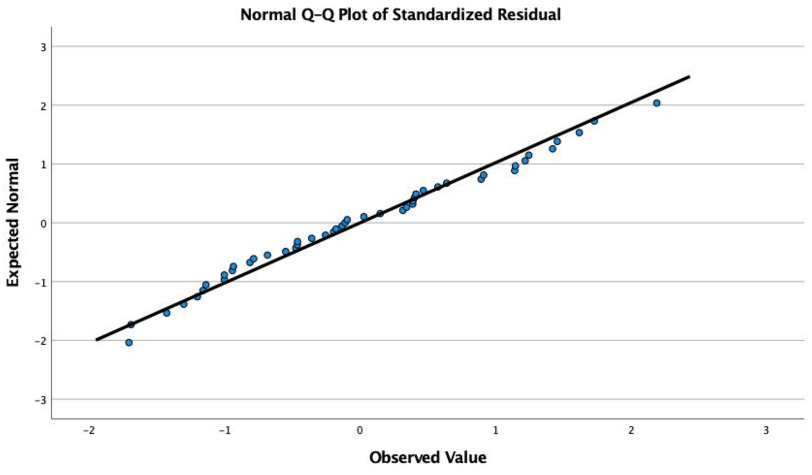

ODI rSLF1 as a function ANT Alertness

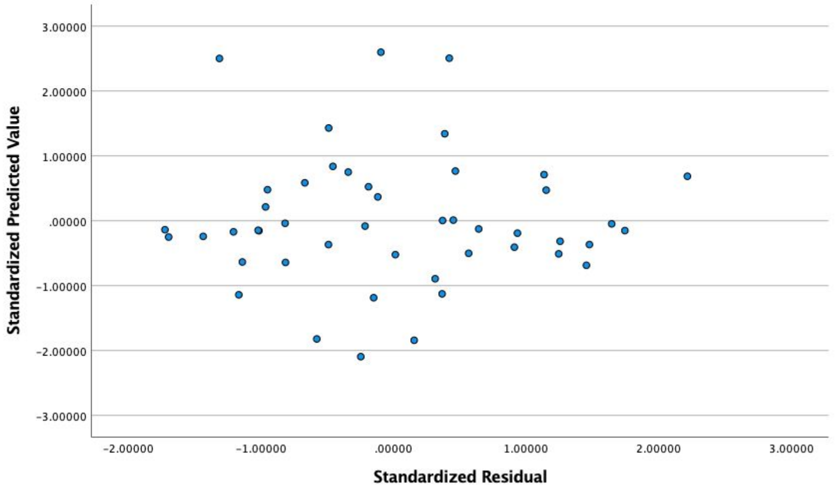

Shapiro-Wilk test of the residuals: W(47)=.98, *p*=.44

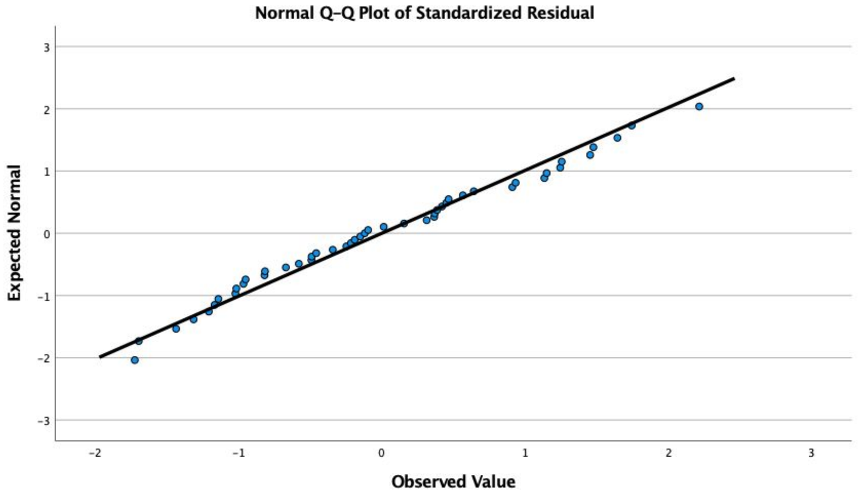

Supplement: fcac080_Supplementary_Data [file Original Submission - Response_to_Reviewers.pdf › Original Submission - Response_to_Reviewers.pdf]
