# Supplementary material for: Right fronto-parietal networks mediate the neurocognitive benefits of enriched environments
Source: Brain Commun. 2022 Mar 28;4(2):fcac080. doi: 10.1093/braincomms/fcac080 (PMC9035529; doi:10.1093/braincomms/fcac080)
Supplement: fcac080_Supplementary_Data [file Revision 1-Decision Letter.pdf › Revision 1-Decision Letter.pdf]

Revision 1- Decision Letter

Dear Dr. Chechlacz,

It is a pleasure to accept your manuscript entitled "Right fronto-parietal networks mediate the neurocognitive benefits of enriched environments" for publication in Brain Communications. Any comments made by the reviewer(s) are included below.

The next step is for your paper to go through "First Look". We will contact you again shortly if any final files or formatting changes are required for publication or if we have any queries.

If no changes are needed, your submission will be transferred to our Production Department and you will hear from the Production Editor in due course.

Video abstracts - Brain Communications encourages authors to provide a video abstract to accompany their article. These are hosted on the Brain/Brain Communications YouTube channel. If you are interested in producing a 5-min video abstract to promote your article, then please contact the Editorial Office (brcoms.editorialoffice@oup.com) for further details and guidelines.

Thank you very much for your contribution. On behalf of the Editors of Brain Communications, we look forward to your future contributions to the Journal.

Best wishes

Dr. Grega Repovs
Associate Editor, Brain Communications

Professor Tara Spires-Jones
Founding Editor, Brain Communications

Reviewer(s)' Comments to Author:

Reviewer: 1 (Helena Gellersen)

Comments to the Author
I commend the authors for expanding on their statistical analyses and adding further sections to the discussion that provide further insight into the interpretation and implications of their findings. The authors have addressed all my comments appropriately. I realise this was a substantial amount of work and I am glad to see that the addition of these statistical approaches has strengthened the results and has provided even more support for the authors' hypothesis regarding the association between EE and SLF branches being specific to SLF1.

I only have a few points to address. This notation: (R2change =.12, Fchange=5.86, p Fchange =.02, unstandardized beta =-588.41, SE=252.99, standardized beta =-.339, 95% CI [-1078.12 - 98.69], Cohen's F2=.16), where the CI for the unstandardised beta follows the standardised beta may be a bit confusing to readers. It might be better to have the CIs follow the unstandardised beta. Moreover, adding p-values to the indirect effect would be beneficial (so far I only see them for the direct effect), even though, of course, the CIs themselves also provide good support for the mediation. Also, line 57 on page 16 should read "Previous theoretical as well as experimental work suggests…"; line 57 on page 19 should read "right hemisphere SLF is strengthened". I trust that all of these points only require quick fixes that can be done during the proofing stage and will not require a formal request to revise the manuscript. I am therefore pleased to accept the revisions and support publication of this manuscript in Brain Communications. I hope this study will have a great impact in paving the way to future exciting hypothesis-driven investigations into the nature of cognitive reserve. Congratulations to the authors for a well-conducted study with very compelling evidence!

Reviewer: 2 (An Vo)

Comments to the Author
The authors addressed issues raised by the Reviewer in the revised manuscript.

Reviewer: 3 (Seraphina Solders)

Comments to the Author
The authors have satisfactorily addressed my previous comments. Their modifications based on other reviewer comments and adjustments to the discussion have greatly improved the manuscript quality, and I am excited to see this work published.
